# Differentiation of endospheric microbiota in ancient and modern wheat cultivar roots

**DOI:** 10.1002/pei3.10062

**Published:** 2021-10-19

**Authors:** Solène Mauger, Claire Ricono, Cendrine Mony, Vèronique Chable, Estelle Serpolay, Marine Biget, Philippe Vandenkoornhuyse

**Affiliations:** ^1^ Université de Rennes 1 CNRS UMR6553 ECOBIO Rennes Cedex France; ^2^ Agrocampus Ouest INRA UMR BAGAP Rennes France

**Keywords:** breeding effect, cultivar effect, microbial recruitment, root endosphere, wheat microbiota

## Abstract

Modern plant breeding and agrosystems artificialization could have altered plants’ ability to filter and recruit beneficial microorganisms in its microbiota. Thus, compared to modern cultivars, we hypothesized that root‐endosphere microbiota in modern wheat cultivars are less resistant to colonization by fungi and bacteria and thus more susceptible to also recruit more pathogens. We used an in‐field experimental design including six wheat varieties (three ancient vs. three modern) grown in monoculture and in mixture (three replicates each). Endospheric microbiota of wheat roots were analyzed on four individuals sampled randomly in each plot. Composition‐based clustering of sequences was then characterized from amplicon mass‐sequencing. We show that the bacterial and fungal microbiota composition in wheat roots differed between ancient and modern wheat cultivar categories. However, the responses observed varied with the group considered. Modern cultivars harbored higher richness of bacterial and fungal pathogens than ancient cultivars. Both cultivar types displayed specific indicator species. A synergistic effect was identified in mixtures of modern cultivars with a higher root endospheric mycobiota richness than expected from a null model. The present study shows the effect of plant breeding on the microbiota associated plant roots. The results call for making a diagnosis of the cultivar's endospheric‐microbiota composition. These new results also suggest the importance of a holobiont‐vision while considering plant selection in crops and call for better integration of symbiosis in the development of next‐generation agricultural practices.

## Graphical abstract

Root endosphere microbiota composition differed among modern (M) and ancient (A) wheat cultivars considering both bacteria (a) and fungi (b)

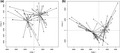

## 1 INTRODUCTION

There is increasing awareness of the opportunity to exploit biodiversity to ensure sustainable livelihoods, food security, and nutrition. Biodiversity increases resilience to shocks and stresses. Diversified farming systems provide opportunities to mitigate emerging challenges and to increase production in a sustainable way (FAO, 2019). Current monocultural agriculture faces a key challenge: the need for sustainability despite limited inputs and fewer environmental impacts and at the same time, for agriculture to be highly productive to limit deforestation in the tropics (Tilman et al., 2011). Among the possible strategies to reach this goal is to make a better use of symbioses in agriculture (e.g. Andreote & Silva, 2017; Bender et al., 2016; Duhamel & Vandenkoornhuyse, 2013). Plants rely to a large extent on the functional efficiency of several symbiotic microorganisms with which they are associated, not only limited to arbuscular mycorrhizal fungi, and which together comprise plant‐microbiota (e.g. Vandenkoornhuyse et al., 2015). For instance, arbuscular mycorrhizal fungi play a tremendous role in crop production because of their importance for plant mineral nutrition (Marx, 2004). Members of the plant microbiota provide a variety of beneficial services that enable plant survival and growth. These include nutrient acquisition, buffering of environmental stresses including drought, protection against soil‐borne pathogens by eliciting plant immune responses (e.g. Berendsen et al., 2012; Vandenkoornhuyse et al., 2015; Vannier et al., 2019), together providing what is termed soil fertility ecosystem services (Guo et al., 2020). However, these microorganisms have not often been incorporated in agricultural management strategies (Bender et al., 2016).

One predicted threat in current agriculture is plant breeding (Duhamel & Vandenkoornhuyse, 2013). The main aim of current breeding is to select the plants that are best adapted to current agricultural practices (Duhamel & Vandenkoornhuyse, 2013). The consequences of this strategy for plant microbiota are poorly documented (Pérez‐Jaramillo et al., 2017) but are expected to impact a plant's ability to select for the most beneficial microorganisms (Valente et al., 2020). Studies have shown that plants can enforce cooperation with microbiota by preferential C allocation to the best symbionts (Kiers et al., 2003, 2011). However, in modern soybean crops, it has also been shown that the ability to filter the good from the bad cooperators has been reduced; modern crops are no longer able to sanction *Bradyrhizobium* cheaters (i.e. defective in nitrogen fixation; Kiers et al., 2007). This was interpreted as a consequence of considering plants as standalone entities in breeding approaches (Duhamel & Vandenkoornhuyse, 2013), leading to the selection of plants that are less dependent on the efficiency of their microbiota (Martin‐Robles et al., 2018; Valente et al., 2019). In addition, conventional agricultural practices have led to a strong genetic homogenization of crops in agricultural landscapes (e.g. Haudry et al., 2007). For nearly 20 years, plant breeding strategies have been engaged in recovering crop diversity using on‐farm and participatory projects in response to the need for the agroecological transition (Chable et al., 2020). Increasing crop diversity, including mixing different cultivars in the same field, has been identified as a key to ensuring crop resistance to environmental stresses including herbivore and pathogen damage (Cheatham et al., 2009; Wuest et al., 2021). However, the importance of such diversity for conservation of the microbial compartment has so far been overlooked. If different genotypes correspond to partially different microbiota, increasing diversity in crop production at local scale (i.e. within a field) should enrich the total pool of recruitable microorganisms in the soil. This potential enrichment may interact with the ability of plants to filter for their symbionts as this would correspond to a larger number of both pathogens and beneficial microorganisms available for selective recruitment.

In this study, we used wheat as a model crop to investigate the effect of modern plant breeding strategies on the endophytic microbiota associated with wheat roots: pure lines fulfilling DUS (Distinct, Uniform, Stable) criteria, called “modern varieties” vs populations/landraces traditionally bred on‐farm, called “ancient”). If modern crops have lost their ability to filter good and bad symbionts, we predict (hypothesis 1) an enrichment of microbiota diversity in modern crops because ancient cultivars may be more prone to select a restricted pool of microorganisms compared to recent cultivars and (hypothesis 2) the possibility that mixed diversity in cultivars in the field increases microbiota species richness for both categories of cultivars, modern and ancient. Knowing that microbiota can contain unwanted microorganisms including pathogens (hypothesis 3) less pathogens in ancient wheat cultivars are expected than in modern cultivars if ancient cultivars better filter the composition of its endospheric community.

To address these hypotheses, an outdoor field experiment was conducted with farmers based on a set of ancient and modern wheat cultivars currently used in agriculture, grown as monoculture and as mixtures. We focused on the wheat root endosphere rather than leaves because the phyllosphere is known to be more variable, i.e. more neutral compared to the root endosphere (e.g. Trivedi et al., 2020) and also because the root‐endosphere microbiota contributes to a level of about ~60% of the leaf microbiota composition (Xiong et al., 2021), the last 40% being a random collection of microorganisms.

## 2 MATERIAL AND METHODS

### 2.1 Experimental design

Six cultivars were chosen in collaboration with the farmers who were part of a participatory project. The wheat cultivars used presented interesting agronomical features and are commonly found in organic farming (‘Bladette de Provence’, ‘Saint Priest et le Vernois Rouge’, ‘Redon Roux Pâle’, ‘Pireneo’, ‘Renan’, and ‘Chevalier’). Based on Roussel et al. (2005), ‘Chevalier’, ‘Renan’, and ‘Pireneo’ are considered as modern cultivars, released in 2006, 1990, and 2004, respectively, and ‘Bladette de Provence’, ‘Saint Priest’, and ‘Redon Roux Pâle’ are considered as ancient cultivars (cited in 1810, 1950, and 1901, respectively). The intensive use of the same ‘strain’ in breeding programs in Europe from 1960 had a bottleneck effect and resulted in modern cultivars with less genetic diversity (Roussel et al., 2005). The seeds of the ancient cultivars used in our study were previously obtained from the wheat Biological Resources Center of Clermont‐Ferrand, France, and were multiplied on the farm for several years.

One farm in Le Rheu, Brittany, France, was chosen for the experiment to limit the possibility of blurring results because of high variability in farming practices and/or soil characteristics. The soil was characterized as loamy, with a pH of 6.2 and a low organic matter rate (2.1%), and rich in magnesium (164 mg/kg). This study is part of a participatory research project that brings together researchers and farmers. The experimental plan was consequently adapted to the machines the farmer in the project used to sow their seeds, and the number of cultivars used in the study was limited by the space available for the study in the farmer's fields. The design comprised three blocks with eight treatments corresponding to each of the three modern and three ancient cultivars alone and a mix of the three modern and of the three ancient cultivars. The three modern cultivars were sown in adjacent plots. Each treatment was sown in October 2016 on a 30 m × 3 m plot. The same agricultural management was performed across the plots before and during the wheat growth.

### 2.2 Root sampling

In each of the 24 plots, four wheat plants aged 5 months were randomly sampled in April 2017, during the heading stage. Differences in root morphology could lead to differences in the microbiota composition (e.g. Ianucci et al., 2021; Saleem et al., 2018). To avoid this issue, comparable root‐tips (i.e. similar diameter, shape, and position) were sampled from the wheat plants. Roots were immediately thoroughly washed first using tap water to remove any remaining soil and then in a 0.5% (w/v) Triton X100 solution for 10 min to remove all microorganisms adhering to the surface of the roots (root epiphytes) (i.e. validated protocol in Lê Van et al., 2017). We did not notice differences in apparent root traits between cultivars. The roots were then rinsed in pure water and cut into ~1 cm pieces. From each individual root sample, we then carefully removed 1–1.5 g of wet roots to represent the whole sample. The root pieces selected were chosen similarly across all the samples. Cleaned roots were dried on Kimwipes and then stored in a 2 ml microtube at −20℃ until root total DNA extraction.

### 2.3 DNA extraction and amplicon sequencing

Roots were ground to powder in liquid nitrogen using a sterile pestle and mortar. DNA samples were extracted using the DNeasy Plant Mini kit 250 (Qiagen) according to the manufacturer's instructions. Fungi and bacteria were targeted using 18S rRNA and 16S rRNA primers, respectively.

The 18S rRNA amplifications were carried out using fungal primers NS22b (5′‐AATTAAGCAGACAAATCACT‐3′) and SSU817 (5′‐TTAGCATGGAATAATRRAATAGGA‐3′) (Lê Van et al., 2017) which enabled us to specifically amplify the fungal DNA of the different known phyla while not amplifying the plastid 18S rRNA (Lê Van et al., 2017). These primers have already been amplified to describe a 530‐bp region of the 18S rRNA gene including the V4 and V5 regions of the 18S rRNA (Borneman & Hartin, 2000). The 16S rRNA amplifications of the V5–V7 region were done using bacterial primers 799F (5′‐AACMGGATTAGATACCCKG‐3′) and 1223R (5′‐CCATTGTAGTACGTGTGTA‐3′) (Vannier et al., 2018). All the primers were modified to include unique sample tag and Illumina® adaptors. The PCR were performed with Illustra™ PuReTaq Ready‐to‐go beads (GE Healhcare®).

Fungal PCR conditions consisted of an initial denaturation step at 95℃ for 4 min followed by 35 cycles at 95℃ for 30 s, 54℃ for 30 s, 72℃ for 1 min, and a final extension step at 72℃ for 7 min. Bacteria PCR conditions consisted of an initial denaturation step at 94℃ for 4 min followed by 35 cycles of 94℃ for 30 s, 53.5℃ for 30 s, 72℃ for 1 min, and a final extension step at 72℃ for 10 min. All PCR products were then purified with AMpureXP magnetic beads (Agencourt®) using an automated liquid platform (Bravo‐Agilent®) and quantified (Quant‐iT PicoGreen™ dsDNA Assay Kit) to allow normalization at the same concentration.

A second PCR was performed using the Smartchip‐Real Time PCR machine (Takara) to achieve multiplex tagging (up to 384 amplicons in one step PCR), the resulting tagged‐amplicon pool was purified (AMpureXP, Agencourt®) and quantified using Kapa Library Quantification Kit‐Illumina® platforms (KAPABIOSYSTEMS®) on a LC480 LightCycler qPCR instrument (Roche®). Pair‐End 2 × 250 and 2 × 300 cycle sequencing runs (MiSeq Instrument, Illumina) were performed on bacterial and fungal sequencing libraries, respectively. Both library preparation and sequence production were performed by the EcogenO Platform (Rennes, France).

### 2.4 Sequence data preparation

Preliminary data trimming consisted in several bioinformatics steps: base‐calling to get reads (fastq), primer removal using the Cutadapt software, and deletion of reads containing unidentified bases. The FROGS pipeline was used to analyze the quality of the filtered sequences (Escudié et al., 2018) with a particular pre‐processing step for fungi (Kozich et al., 2013) and the standard protocol for bacteria reads. The FROGS pipeline produces sequence clusters using SWARM (Escudié et al., 2018), which enables group reads without using identity threshold and is thus closer to ASV analysis than to standard OTUs. One of the known advantages of FROGS is to limit or avoid the overestimation of the true microbiota composition related to the multicopy status bias of the SSU rRNA gene in bacteria and fungi and possible variants existing within an individual. A contingency matrix was produced using highly stringent processes as recommended by Escudié et al. (2018). SILVA 123 databases (16S and 18S) (Quast et al., 2013) were used for sequence clustering. Sequence clusters were filtered using the quality of the affiliations with a threshold of at least 95% coverage and 95% BLAST identity.

Datasets were normalized at 20,000 and 15,000 sequences per sample for bacteria and fungi, respectively. A diversity index (Pielou's evenness and sequence‐cluster richness) was calculated using the R package “vegan” (Oksanen, 2019). Pearson's correlation between the two indices was calculated and was below 0.70 (Dormann et al., 2013). Sequences are available from the ENA accession number PRJEB37900.

### 2.5 Pathogen identification

Known plant fungal pathogens in the root‐fungal microbiota were identified from the literature and MycoBank (MycoBank, http://www.mycobank.org; Crous et al., 2004) and double‐checked in the FUNGuild database (Nguyen et al., 2016). Only Families including widespread plant pathogen species such as Helicobasidium, Magnaporthaceae, and *Microascales* were taken into account.

### 2.6 Statistical analyses

Analyses were performed of whole bacteria and fungi matrixes and also on subsets of the whole dataset corresponding to the most abundant groups of sequence clusters (i.e. Proteobacteria, Bacteroidetes, Actinobacteria, Acidobacteria, Firmicutes for the bacteria; Ascomycetes, Basidiomycetes, Glomeromycetes, and Chytridiomycetes for the fungi).

We used Partial Least Square‐Discriminant Analyses (PLS‐DAs) to analyze the effect of the category of cultivar (ancient vs. modern) on sequence‐cluster composition (i.e. β‐diversity analysis). On Hellinger's transformed data, PCoA analyses have also been done to confirm the results and are now provided as Supporting information (see below). In contrast with PLS‐DA, these PCoA analyses are not constrained and differences in composition among samples can be tested afterward by permutation tests. Thus, Permutational Multivariate Analysis of Variance (Adonis) to compare root‐endosphere microbial communities in modern versus ancient wheat cultivars was performed. To test hypothesis 1, enrichment of microbiota diversity in modern crops in comparison to ancient, we then calculated the percentage of sequence‐clusters shared by ancient and modern cultivars based on a presence threshold of 100%, 95%, 90%, and 50% of the samples. We used linear mixed models to analyze the effect of variety and of the category of cultivar (ancient vs. modern) on sequence‐cluster richness and evenness both for fungi and bacteria and at the phylum level. These analyses were only performed on varieties that were grown alone. Lastly we determined sequence‐clusters that were indicators of each of the two cultivar categories (i.e. ancient and modern cultivars). A permutation test allowed to calculate a P value to identify the ‘indicator species’, i.e. particular sequence clusters occurring more often in the wheat root microbiota of old *versus* modern cultivars and reciprocally. More information about the statistical inference is provided in De Cáceres et al. (2010). The ‘Indicators species’ analysis was performed using the indicspecies package in R (De Cáceres et al., 2010).

The linear models were chosen to account for nested random factors induced by the experimental patterns. The random factors used for models to test the variety effect were block cultivar category (i.e. ancient vs modern)/replicate/position of the cultivar. The random factors used for models to test the effect of cultivar category were replicate/position of the cultivar. Each model was analyzed using ANOVA. The distribution of residual normality was checked graphically for each model to respect ANOVA normality conditions, *R* ^2^ was calculated to determine the amount of variance explained by the different models, and a significance threshold of *p* < 0.05 was chosen. Marginal (*R* ^2^m) and conditional (*R* ^2^c) *R* ^2^ values were calculated, corresponding to variance explained by fixed effect or fixed and random effects respectively. Mixed linear models were performed with the lme4 (Bates et al., 2015), nlme (Pinheiro et al., 2020), car (Fox & Weisberg, 2019), MuMIn (Barton, 2019), and lmer (Kuznetsova et al., 2017) packages.

To test hypothesis 2, the possibility that mixed diversity in cultivars in the field increases microbiota species richness for both categories of cultivars, modern and ancient, we calculated the values for sequence‐cluster richness and evenness, for each mixture while expecting an additive effect of the three cultivars mixed evenly grown together. The expected value was the mean of the sequence‐cluster richness or the sequence‐cluster evenness of the cultivars from the monocultures. Expected values were calculated by random sampling within the values among each type of treatment. We tested if observed sequence‐cluster richness or evenness in mixtures differed from expected values using a *t*‐test (12 observed values compared to 12 randomly chosen expected values). If significant, positive difference demonstrated a synergistic effect while negative difference demonstrated an antagonistic effect of the mixture of cultivars.

To test hypothesis 3 (i.e. less pathogens in root endosphere of ancient cultivars) similar models were used on plant pathogens in the total fungi and bacterial communities. The same analysis was done on the richness and relative abundance of plant pathogens found in the matrix. These analyses were only performed on varieties that were grown alone and not mixed with other cultures.

All statistical analyses were conducted using R Studio version 1.3.959 (R Core Team, 2016).

## 3 RESULTS

### 3.1 Microbial community description

**FIGURE 1:**
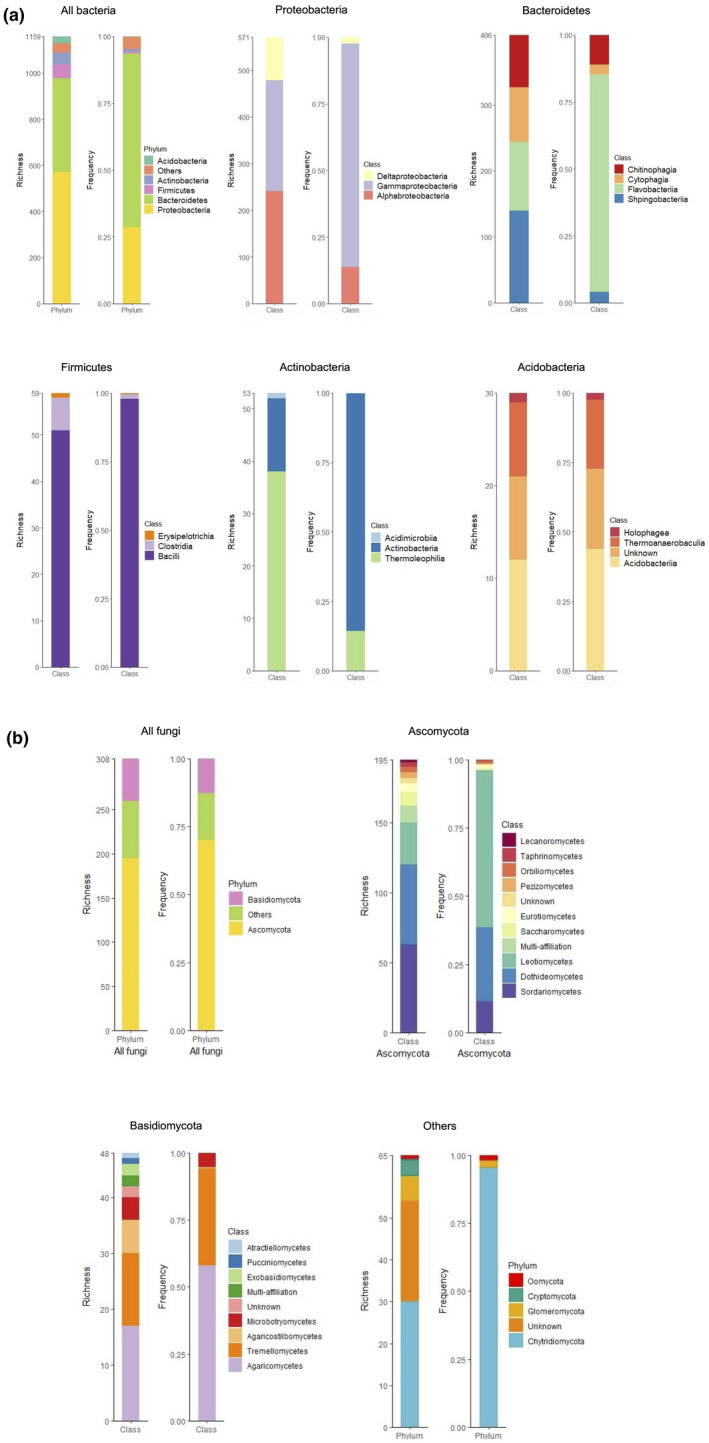
1 Sequence‐cluster richness and relative abundance of the wheat root endospheric bacterial (a) and fungal (b) microbiota (γ‐diversity thus not considering the wheat cultivar) and within the most widely represented phyla

At the γ‐diversity scale (i.e. total microbiota diversity), the wheat root endospheric bacterial community was mainly composed of Proteobacteria (571 sequence‐clusters, 28% of the sequences) and the Bacteroidetes (406 sequence‐clusters, 65% of the sequences) phyla (Figure 1). Firmicutes, Actinobacteria and Acidobacteria were less represented with a richness of 59, 53, and 30 sequence‐clusters respectively (Figure 1). Considering fungi, the wheat endospheric microbiota was composed of 70.0% of Ascomycota (195 sequence‐clusters), 12.8% of Basidiomycota (48 sequence‐clusters), and 17.3% of Chytridiomycota and Glomeromycota (65 sequence‐clusters) (for more details on the community composition, see Figure 1).

### 3.2 Effect of wheat cultivar category on root‐endospheric microbiota assemblages

**FIGURE 2:**
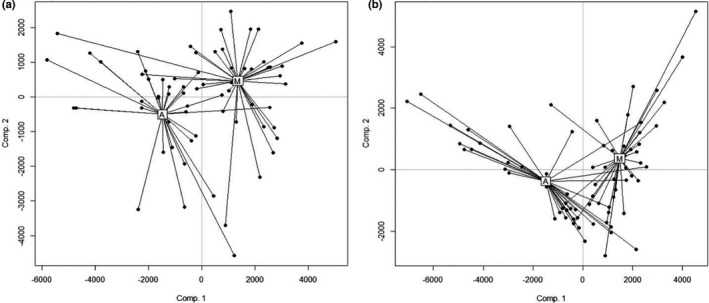
Partial Least Squares Discriminant Analysis (PLS‐DA) ordination of the bacterial (a) and fungal (b) community in ancient (A) and modern (M) cultivars tested on the total community (1159 sequence‐clusters for the bacteria and 308 for the fungi)

The microbiota community composition was compared in samples of ancient and modern cultivars. The PLS‐DA ordinations revealed significant modification of the bacterial and fungal community composition colonizing the roots between ancient and modern wheat cultivars (*P* PLS‐DA = 0.001, *P* modern vs ancient <0.01) (Figure 2; Table S1), confirmed by the PCoA analyses (Figure S1, Table S2). This analysis was also performed at the phylum level for fungal and bacterial communities. Except for Actinobacteria, the same significant pattern was observed at the level of the phyla (Table S1).

The microbiota composition differed considerably between ancient and modern wheat cultivars. Less than 4% of the bacterial or fungal sequences were shared between the ancient and modern cultivars (i.e. 3.3% of bacteria and 3.6% of the fungi), considering all the samples (Table 1). When considering less stringent thresholds, these differences decreased rapidly for bacteria to nearly 100% of similarity among ancient and modern cultivars when considering only 50% of the samples. On the contrary, the differences between ancient and modern cultivars in fungi composition were maintained even at the 50% threshold. The ‘indicator species’ analyses demonstrated strong specificities between modern and ancient cultivar categories. We detected 168 bacterial and 29 fungal sequence‐clusters that were significantly more abundant in modern wheat cultivars while 256 bacterial and 16 fungal sequence‐clusters were significantly more abundant in ancient compared to modern cultivars explaining at least in part the differentiation observed among modern and ancient wheat microbiota composition (Figure 2). Within the sequence‐clusters that were indicators of one given cultivar category, modern cultivar displayed more Gammaproteobacteria than ancient cultivars, while ancient cultivars displayed more Actinobacteria (dominated by Thermoleophilia), Alphaproteobacteria, Deltaproteobacteria (dominated by Bdellovibrionales) than modern wheat cultivars (Table S3). Some such as Acidobacteria and Gemmatimonadetes were ‘indicator species’ of the ancient cultivars, and Fibrobacteres of modern cultivars. It can also be noticed that within the Firmicutes, sequence clusters affiliated to Paenibacillaceae and Bacillales were indicators of modern and ancient cultivars microbiota, respectively. In fungi, there were also ‘indicator species’ of both cultivar categories, notably more Ascomycota (dominated by Sordariomycetes) in modern cultivars while more Basidiomycota (dominated by Tremellomycetes) associated with ancient cultivars. Glomeromycota and Chytridiomycota contained indicator species of modern cultivars only (Table S3).

**TABLE 1:** Rate of common sequence‐clusters (%) between ancient and modern wheat cultivars within all groups of bacteria and fungi and for the most abundant phyla

Group	100%	95%	90%	50%
All bacteria	3.3	30.7	49.4	99.1
Proteobacteria	3.1	28.5	48.3	99.5
Bacteroidetes	4.2	37.8	55	99.5
Actinobacteria	3.8	17	34	98.1
Firmicutes	0	25.4	42.4	93.2
Acidobacteria	0	13.3	40	100
All fungi	3.6	4.2	5.8	11.7
Ascomycetes	3.6	4.1	5.6	12.8
Basidiomycetes	6.3	8.3	8.3	14.6
Other fungi	1.5	1.5	3.1	6.2

This rate was calculated for different thresholds (100%, 95%, 90% and 50%). Rates at 100% correspond at sequence‐clusters that are common for 100% of replicates between ancient and modern wheat cultivars. Rates at 50% correspond at sequence‐clusters that are common for at least 50% of replicates among ancient and modern wheat cultivars.

Beside the detected ‘indicator species’ described above, the number of sequence clusters was significantly higher in the ancient cultivars for Actinobacteria (*p* < 0.001), while higher in the modern cultivars for the Firmicutes phylum (*p* = 0.0013; Table 2). The cultivar ‘Bladette de Provence’ provided a higher richness of Bacteroidetes than the cultivar ‘Redon Roux Pâle’. The cultivar ‘Redon Roux Pâle’ provided a higher richness of Acidobacteria than the cultivar ‘Pireneo’. The effect of cultivar categories on sequence‐cluster richness was detected in two out of the three classes studied, and also for the richness of all fungi. Considering the fungal microbiota, apart from the Basidiomycota (NS), the number of sequence clusters was higher in the roots of modern cultivars (‘All fungi’ *p* = 0.002; ‘Ascomycota’ *p *= 0.01; ‘other phyla’ *p *< 0.001; Table 2).

**TABLE 2:** Effect of the category of wheat cultivar; Ancient (A) or Modern (M); and of the wheat cultivar Bladette de provence (BL), Redon Roux Pâle (RD), Saint Priest et le Vernois Rouge (SP), Renan (“RE)”, Pireneo (PI), and Chevalier (CH) on the number of bacterial and fungal sequence clusters measured with a linear mixed model

Linear mixed model on the sequence‐ cluster richness matrix of:	Variable	*R* ^2^m	*R* ^2^c	*χ* ^2^	*p*	Output
All bacteria	Cultivar category	0.018	0.141	0.98	0.322	—
	Cultivar	0.101	0.372	8.99	0.109	—
Proteobacteria	Cultivar category	0.032	0.176	1.74	0.187	—
	Cultivar	0.084	0.361	6.14	0.293	—
Bacteroidetes	Cultivar category	0.0002	0.254	0.0002	0.98	—
	Cultivar	0.096	0.47	11.66	0.039^*^	BL > RD
Acidobacteria	Cultivar category	0.048	0.048	3.38	0.066	—
	Cultivar	0.19	0.36	15.36	0.0089^**^	RD > PI
Actinobacteria	Cultivar category	0.19	0.19	16.01	<0.001^***^	A > M
	Cultivar	0.206	0.345	6.51	0.2597	—
Firmicutes	Cultivar category	0.15	0.21	10.27	0.001^**^	M > A
	Cultivar	0.18	0.37	7.46	0.1884	—
All fungi	Cultivar category	0.11	0.2	9.91	0.002^**^	M > A
	Cultivar	0.12	0.34	4.11	0.53	—
Ascomycota	Cultivar category	0.08	0.13	6.77	0.01^**^	M > A
	Cultivar	0.13	0.28	6.41	0.27	—
Basidiomycota	Cultivar category	0.02	0.02	0.28	0.28	—
	Cultivar	0.03	0.22	1.55	0.91	—
Other fungi	Cultivar category	0.19	0.37	17.24	<0.001^***^	M > A
	Cultivar	0.21	0.46	7.85	0.16	—

*R* ^2^m and *R* ^2^c are marginal and conditional *R* ^2^ values, corresponding to variance explained by fixed effect or fixed and random effects respectively.

*** means very highly significant, ** means highly significant, and * means statistically significant.

Microbial community structure differed among cultivars. The evenness of total bacteria was significantly higher (*p *= 0.0155) and of the Bacteroidetes phylum (*p *< 0.001) in modern cultivars, while the ancient varieties had a higher Pielou's index of Proteobacteria (*p* = 0.0014) and Actinobacteria (*p *< 0.001; Table 3). The structure of the fungal community also differed among cultivars. The evenness descriptor of Basidiomycota was significantly higher in ancient varieties (*p *< 0.001), but that of total fungi was significantly higher (*p *< 0.001), Ascomycota (*p *< 0.001), and the other phyla (*p *< 0.01) in the roots of modern cultivars (Table 3).

**TABLE 3:** Effect of the category of wheat cultivar Ancient (A) or Modern (M) and of the wheat cultivar ‘Bladette de provence’ (BL), ‘Redon Roux Pâle’ (RD), ‘Saint Priest and Vernois Rouge’ (SP), ‘Renan’ (RE), ‘Pireneo’ (PI), and ‘Chevalier’ (CH) on bacterial and fungal sequence‐clusters evenness measured with a linear mixed model

Linear mixed model on the evenness matrix of:	Variable	*R* ^2^m	*R* ^2^c	*χ* ^2^	*p*	Output
All bacteria	Cultivar category	0.081	0.085	5.86	0.016^*^	M > A
	Cultivar	0.134	0.292	7.18	0.207	—
Proteobacteria	Cultivar category	0.16	0.28	10.22	0.0014^**^	A > M
	Cultivar	0.149	0.37	3.37	0.64	—
Bacteroidetes	Cultivar category	0.17	0.17	13.96	<0.001^***^	M > A
	Cultivar	0.25	0.45	15.78	0.0075^**^	—
Acidobacteria	Cultivar category	0.004	0.004	0.26	0.61	—
	Cultivar	0.037	0.21	2.8	0.73	—
Actinobacteria	Cultivar category	0.203	0.241	15.38	<0.001^***^	A > M
	Cultivar	0.275	0.453	14.75	0.0115^*^	RE > PI
Firmicutes	Cultivar category	0.012	0.051	0.82	0.3628	—
	Cultivar	0.075	0.24	5.88	0.3176	—
All fungi	Cultivar category	0.17	0.31	10.8	<0.001^***^	M > A
	Cultivar	0.29	0.43	19.8	0.001^**^	BL < PI
Ascomycota	Cultivar category	0.28	0.47	15.74	<0.001^***^	M > A
	Cultivar	0.31	0.55	8.68	0.12	—
Basidiomycota	Cultivar category	0.17	0.2	13.77	<0.001^***^	A > M
	Cultivar	0.15	0.34	3.41	0.6366	—
Other fungi	Cultivar category	0.14	0.25	8.51	<0.01^**^	M > A
	Cultivar	0.13	0.39	2.63	0.76	—

*R* ^2^m and *R* ^2^c are marginal and conditional *R* ^2^ values, corresponding to variance explained by fixed effect or fixed and random effects respectively.

*** means very highly significant, ** means highly significant, and * means statistically significant.

If obvious differences are found among root‐endosphere microbiota composition (Figure 2, Tables 2 and 3), this effect could not be attributable to a particular cultivar displaying a divergent microbiota (Tables 2 and 3). If little effects are detected at the cultivar level, this is likely related to both heterogeneity in the microbiota composition for a given cultivar and more limited statistical power (i.e. number of corresponding replicates).

### 3.3 Additive effect of the mixture

By comparing the expected and observed richness and evenness of the wheat root‐microbiota either mixing the three ancient or the three modern cultivars, we aimed to test the null hypothesis of additivity of each cultivar independently.

Considering bacteria, the null hypothesis of additivity of both microbiota composition and microbiota community structure cannot be rejected (NS) for the ancient cultivars. Conversely, the observed fungal microbiota richness in modern cultivars was higher than just an additivity phenomenon, for most of the fungi (Table 4). Apart from Proteobacteria, the observed bacterial community structure measured by Pielou's evenness did not differ from the expected results under the null hypothesis of additivity in the roots of modern cultivars. The evenness pattern within fungi varied in ancient and modern cultivars depending on the fungal phyla (Table 4).

**TABLE 4:** Comparison of expected richness and evenness in a mixed culture under the null model of additivity (Exp) and observed richness and evenness when the three cultivars were grown together (Obs)

	Null model additivity on sequence‐clusters richness						Null model additivity on sequence‐clusters evenness					
	Modern			Ancient			Modern			Modern		
	df	*p*	Variable	df	*p*	Variable	df	*p*	Variable	df	*p*	Variable
All bacteria	23.9	0.17	—	21.29	0.39	—	18.5	0.58	—	23.9	0.63	—
Proteobacteria	22	0.059	—	21.3	0.53	—	21.2	0.003^**^	Exp >Obs	21.9	0.17	—
Bacteroidetes	21.2	<0.001^***^	Obs >Exp	17.3	0.88	—	17.9	0.93	—	21.8	0.99	—
Acidobacteria	21.9	0.0285^*^	Exp >Obs	13.9	0.59	—	17.4	0.56	—	21.9	0.80	—
Actinobacteria	21.9	0.4198	—	20.8	0.28	—	16.3	0.26	—	12.5	0.06	—
Firmicutes	14.7	0.2564	—	21.3	0.82	—	21.9	0.17	—	20.7	0.20	—
Other bacteria	20.3	0.002^**^	Obs >Exp	19.8	0.90	—	18.7	0.85	—	21.3	0.77	—
All fungi	17.7	<0.001^***^	Obs >Exp	18.02	0.85	—	18.0	0.27	—	14.4	0.25	—
Ascomycota	20.8	<0.001^***^	Obs >Exp	14.42	0.29	—	20.6	0.002^**^	Exp >Obs	13.9	0.002^**^	Obs >Exp
Basidiomycota	15.5	0.004^**^	Obs >Exp	20.35	0.71	—	20.0	<0.001^***^	Obs >Exp	16.0	0.143	—
Other fungi	22.0	0.126	—	18.2	0.34	—	19.1	0.117	—	15.8	0.003^**^	Obs >Exp

The microbiota in the ancient and modern cultivars are compared at different levels (rows in the table). The *p*‐values were obtained using a *t*‐test.

*** means very highly significant, ** means highly significant, and * means statistically significant.

### 3.4 Effect of the category of cultivar on pathogens

The modern cultivar categories had a significantly higher number of pathogenic bacteria and fungi in their root microbiota endosphere (respectively *p *= 0.004 and *p* = 0.015 respectively, Table 5), higher pathogenic bacteria relative abundance (*p* < 0.001, Table 5). Strikingly, the pathogenic bacteria richness was higher than expected when the ancient cultivars were mixed with modern cultivars (Table 6).

**TABLE 5:** Effect of the category of wheat cultivar, Ancient (A) or Modern (M), on the pathogenic bacterial and fungal richness and relative abundance detected in the host‐plant microbiota

Group	Variable	Richness					Relative abundance				
		*R* ^2^m	*R* ^2^c	*χ* ^2^	*p*	Output	*R* ^2^m	*R* ^2^c	*χ* ^2^	*p*	Output
Bacteria	Cultivar category	0.13	0.22	8.47	0.0036^**^	M>A	0.13	0.39	24.74	<0.001^***^	M > A
	Cultivar	0.19	0.43	10.54	0.0613	—	0.28	0.45	5.35	0.36	—
Fungi	Cultivar category	0.11	0.28	5.81	0.015^*^	M>A	0.045	0.36	1.71	0.19	—
	Cultivar	0.12	0.45	4.18	0.5234	—	0.13	0.48	5.66	0.34	—

The results were obtained from a linear mixed model.

*** means very highly significant, ** means highly significant, and * means statistically significant.

**TABLE 6:** Comparison of both expected pathogen richness and evenness in a mixed culture under the null hypothesis of additivity (Exp) and observed richness or evenness of each pathogen when the three cultivars were grown together (Obs), in ancient and modern categories of wheat

Group	Richness						Abundance					
	Modern			Ancient			Modern			Ancient		
	df	*p*	Output	df	*p*	Output	df	*p*	Output	df	*p*	Output
All bacteria	16.9	0.003^**^	Obs >Exp	14.5	0.92	—	17.9	0.94	—	21.3	0.44	—
Proteobacteria	22.0	<0.001^***^	Obs >Exp	17.6	0.33	—	20.9	0.64	—	19.0	0.76	—
Firmicutes	22.0	0.37	—	15.1	0.79	—	17.2	0.13	—	18.1	0.88	—
Actinobacteria	18.1	0.74	—	18.1	0.28	—	17.6	0.66	—	14.0	0.002^**^	Obs >Exp
All fungi	20.2	0.67	—	16.1	0.84	—	21.5	0.85	—	11.6	0.36	—

The *p*‐values were obtained using a *t*‐test.

*** means very highly significant, ** means highly significant, and * means statistically significant.

## 4 DISCUSSION

A total of 1159 and 308 sequence‐clusters of bacteria and fungi respectively were detected in wheat roots sampled in the field experiment. We demonstrated major changes in sequence‐cluster composition associated with wheat depending on the category of cultivar (Figure 2: PLS‐DA). Less than 4% of the sequence‐clusters were present in all the samples of ancient and modern cultivars but the bacteria were more similar among wheat categories than fungi.

### 4.1 Microbiota in ancient wheat cultivars differed from those in recent ones

Validating our first hypothesis, we demonstrated that the microbial composition of both bacteria and fungi differed between ancient and modern cultivars (Table 3, Figure 2, Figure S1). Modern cultivars displayed a significantly higher number of fungal sequence‐clusters and a more equitable mycobiota compared to ancient cultivars (Tables 2 and 3) and a more equitable bacterial microbiota (Table 3). However, we cannot exclude the possibility that very fine scale heterogeneity among the experimental sites could have influenced our results.

As expected, ancient cultivars had a smaller pool of root‐endosphere microbiota than modern cultivars, probably due to their increased ability to filter the recruited microorganisms. Such an effect has also been reported in other crops (Bulgarelli et al., 2015). The filtering effect probably results from exudates (rhizodeposits) actively released by plants, and that affect the composition of microorganisms in the rhizosphere (Pérez‐Jaramillo et al., 2016; Sasse et al., 2018). Root exudates could also be dependent on root morphology (Proctor & He, 2017) leading to a differentiated microbiota according to root type (Iannucci et al., 2021). Herein, the sampled roots were comparable for all the wheat‐plant analyzed.

Assuming that the root microbiota endosphere mainly results from the recruitment of rhizosphere microorganisms, a filtering effect would be expected for both bacteria and fungi. We observed that the filtering effect of the root‐endospheric microbiota in ancient wheat cultivars was stronger for fungi than for bacteria (Tables 2 and 3). In ancient cultivars, the observed higher Actinobacteria richness together with lower Firmicutes richness likely blurred the filtering they imposed on the recruitment of their microbiota at the whole bacterial scale. Nevertheless, the filtering effect was also clearly shown through the specific taxa that are indicators of either modern or ancient cultivars. This is true for both fungal‐ and bacterial‐ indicator sequence clusters evidenced.

Changes in wheat root microbiota endosphere were also recorded but to a lesser extent among genotypes in both ancient and modern cultivars, especially bacteria, which differed in the richness of particular phyla among genotypes. Such intra‐group variability could be linked to the breeding history of each cultivar both in terms of age and of the selection objective. Breeding is not linear over time and may thus result in complex patterns (Van de Wouw et al., 2010) that are difficult to link to a given footprint of domestication.

Despite the limited number of cultivars used in this study, our work provides evidence for differentiation among microbial communities colonizing roots of ancient vs modern cultivars. This may indirectly determine the presence and abundance of pathogens in plant microbiota. Assuming that crop yield performance is the main objective of farming, selection by breeders for this aim has led to modern crops that invest less in resource harvesting than ancient cultivars of wild ancestors (Anten & Vermeulen, 2016). If selection is only based on the grain yield trait, an individual in a population that is disease resistant would likely emerge as better than the others if it reduced its investment to the cost of defense. Thus, it has been hypothesized that this selection strategy leads to selection for lower defense (Anten & Vermeulen, 2016). If this is true, this defense payoff could explain why the modern wheat cultivars in our study displayed reduced ability to filter the microorganisms that comprise its microbiota. Thus, an important prospect to develop the understanding of the process at work is to test the possibility that the wheat cultivars have different secreted exudate composition. Another prospect related to the wheat‐cultivars differential microbiota recruitment would be to analyze in addition to the root‐microbiota endosphere, other host‐plants microhabitats including rhizosphere, stem, leaves and seeds.

### 4.2 More pathogens in modern wheat cultivars than in ancient cultivars

In agreement with our working hypothesis (i.e. hypothesis 3), we showed that ancient cultivars had fewer pathogenic bacterial microorganisms than modern cultivars both in terms of species richness and abundance while only an effect on fungal richness was detected. We identified three limitations to this important result. First, the differential effect observed between bacterial and fungal pathogens is likely related to our ability to detect the pathogens and sequence databases. Second, one of the three modern cultivars (i.e. ‘Renan’) was assumed to be ‘resistant’ to *Fusarium*, *Puccina*, and *Tapesia* under certain environmental conditions (Dedryver et al., 2009; Gervais et al., 2003). However, intuitively, we would expect to detect fewer pathogens in the root endospheric microbiota of this ‘resistant’ cultivar, which was not the case. Third, a larger pool of cultivars would have to be tested to confirm our results. Even considering these limitations, the results concerning the pathogen guild confirm the fact that modern cultivars have less ability to filter the microorganisms they recruit within their endosphere (Table 5). This is most likely one result of the said plant defense payoff resulting from the breeding strategy which seems to be a parsimonious explanation for the observation. If this is true, the susceptibility of modern crops to recruit pathogens and parasites is likely widely shared. Because plants leave a footprint of their microbiota in soils (Bittebière et al., 2019), modern crops, even those assumed to be resistant—as was the case in our study—can enrich the pool of soil‐borne pathogens. As already emphasized by Denison (2011), it is important to grow plants that reduce this soil‐borne pathogen pool in agricultural soils.

### 4.3 Is there a synergistic effect of mixing cultivars on microorganisms?

Starting from the idea of synergy, we hypothesized that mixing different wheat cultivars in the field would increase both the richness and diversity of the wheat root microbiota endosphere (i.e. hypothesis 2). This null hypothesis was not rejected in the case of ancient cultivars grown in a mixture considering both bacteria and fungi. Conversely, the synergistic effect is true in the case of the fungal microbiota since the observed richness of the mixed modern cultivars displayed was higher than expected (Table 4). Furthermore, in the parcels of mixed cultivars, a significantly higher number of bacterial pathogens was observed in the roots of modern cultivars (Table 6) but not in the root of ancient ones.

On one hand, an increase in the microbial and fungal reservoir would be expected when ancient cultivars are mixed. On the other hand, because of the reduced ability of modern wheat to filter their root microbiota, a mixture of modern cultivars would increase the total pool of microorganisms but would also inadvertently increase the pool of pathogens and likely that of bad cooperators. Given the fact that neighboring plants directly affect the microbiota of the focal plant (Bittebière et al., 2019; Mony et al., 2021), if ancient wheat cultivars make it possible to mitigate the pool of recruitable pathogens, a mixture of modern/ancient cultivars could limit the increase in the number of pathogens in wheat microbiota communities. However, this kind of mixture has not yet been tested in the field because it was argued that such a mixture would lead to competition between tall and short wheat, not between ancient and modern. More widely, there is growing interest in designing mixtures of cultivars (e.g. Barot et al., 2017), but little has been done with respect to the crop microbiota.

The payoff from investment in resources for filtering pathogens and rewarding/punishing good and bad symbionts could be re‐allocated to grain production if farming practices compensate for the damaged ecological functions. As a result, breeding has unintentionally selected cultivars that could damage the microbial reservoir by not efficiently filtering their microbial colonizers. To our knowledge, this has never been tested to date even though it is highly relevant for agriculture.

### 4.4 Collateral damage caused by current agriculture and the need for more sustainable agriculture

To date, little attention has been paid to symbioses and microorganisms in agriculture despite their fundamental importance for plant survival and reproduction (Vandenkoornhuyse et al., 2015; Wille et al., 2019). Even worse, conventional agriculture is likely damaging key ecological processes including soil fertility ecosystem services (Guo et al., 2020). For instance, a recent study demonstrated that diazotrophy, the ability to breakdown N2 into NH4, is drastically reduced after long‐term nitrogen amendment of agricultural soils (Fan et al., 2019). It is also known that current agricultural practices have damaged both the functional efficiency and diversity of arbuscular mycorrhizal fungi (Verbruggen & Kiers, 2010). It has also been emphasized in soybean that modern cultivars are no longer able to stop defective nitrogen fixers from forming nodules (Kiers et al., 2007). In the same line of thought, here we showed in the field that modern wheat cultivars are less efficient in filtering the microorganisms colonizing roots and are more prone to recruiting pathogens. This pathogen enrichment in modern wheat crops as observed herein might negatively impact (i) other cultivars eventually cultivated/grown in the same area and (ii) the next crops by the legacy on the soil microorganisms reservoir of this pathogen‐enrichment. As far as we know, these hypotheses have never been tested.

The examples developed above show how urgent it is to perform diagnoses to get a clearer picture of the consequences of current agricultural practices, breeding, GMOs and genome edited crops, for symbiotic and pathogenic reservoirs, not only in terms of microbial richness and diversity, but also in terms of functions. Our results also offer new opportunities but need to be confirmed using different wheat cultivars, sampling dates, locations, experimental designs and using other crops than wheat.

## 5 CONCLUSIONS

The root‐endospheric wheat microbiota, the most intimate fraction of the plant‐interacting microorganisms, clearly differs among modern and ancient wheat cultivars (Figure 2). Despite the limited wheat cultivar used herein, is clear that modern wheat cultivars filter less the microorganisms colonizing their roots and seem more prone to recruit pathogens than ancient cultivars. Because these results are new, the study will need to be confirmed for wheat and also possibly for other crops and mechanisms of microbial recruitment to form the root‐microbial endosphere have to be studied.

The idea that ‘Plant breeding has to go microbial’ has been developed in a paper by Wei and Jousset (2017). Our findings lead to the conclusion that a modification of the current plant‐breeding paradigm is likely necessary with plant‐symbiotic microorganisms to be considered. In Agreement to Duhamel and Vandenkoornhuyse (2013), our findings also lead to the conclusion that an exhaustive diagnosis of cultivars used in agriculture for their ability to interact with efficient symbionts is needed.

If we are to achieve sustainable agriculture, breeding strategies will need to be rethought in depth (e.g. Chable et al., 2020; Denison, 2011; Döring et al., 2011; Duhamel & Vandenkoornhuyse, 2013; Hohmann & Messmer, 2017; Lammerts van Bueren et al., 2018; Wei & Jousset, 2017; Wuest et al., 2021), and plants and crops can no longer be regarded as standalone entities but rather as holobionts (the host plus its microbiota) that together form the individual (Vandenkoornhuyse et al., 2015). Far from the prevailing school of thought in mainstream agriculture and plant sciences, we call for a more holistic vision, and the design of new frameworks to search for more sustainable crop and food production.

## CONFLICT OF INTEREST

The authors declare no conflict of interest.

## AUTHOR CONTRIBUTIONS

VC and ES conceived the experiment, SM and MB did the sampling and molecular analyses, SM, CR, CM and PV did the data analyses, SM, CR, CM and PV wrote the paper.

## Supporting information

Supplementary Material

## Data Availability

All the sequence data are available at the European Nucleotide Archive (ENA) under the accession number PRJEB37900.

## References

[pei310062-bib-0001] Andreote, F. , & Silva, M. (2017). Microbial communities associated with plants: Learning from nature to apply it in agriculture. Current Opinion in Microbiology, 37, 29–34. 10.1016/j.mib.2017.03.011. 28437663

[pei310062-bib-0002] Anten, N. P. R. , & Vermeulen, P. J. (2016). Tragedies and crops: Understanding natural selection to improve cropping systems. Trends in Ecology and Evolution, 31, 429–439. 10.1016/j.tree.2016.02.010 27012675

[pei310062-bib-0003] Barot, S. , Allard, V. , Cantarel, A. , Enjalbert, J. , Gauffreteau, A. , Goldringer, I. , Lata, J. C. , Le Roux, X. , Niboyet, A. , & Porcher, E. (2017). Designing mixtures of varieties for multifunctional agriculture with the help of ecology. Agronomy and Sustainable Development, 37, 13. 10.1007/s13593-017-0418-x

[pei310062-bib-0004] Bartoń, K. (2019). MuMin. Multi Model Inference R. 155.232.191.229.

[pei310062-bib-0005] Bates, D. , Maechler, M. , Bolker, B. , & Walker, S. (2015). Fitting linear mixed‐effects models using lme4. Journal of Statistics Software, 67, 1–48.

[pei310062-bib-0006] Bender, S. F. , Wagg, C. , & van der Heijden, M. G. A. (2016). An underground revolution: Biodiversity and soil ecological engineering for agricultural sustainability. Trends in Ecology and Evolution, 31, 440–452. 10.1016/j.tree.2016.02.016. 26993667

[pei310062-bib-0007] Berendsen, R. L. , Pieterse, C. M. J. , & Bakker, P. A. H. M. (2012). The rhizosphere microbiome and plant health. Trends in Plant Science, 17, 478–486. 10.1016/j.tplants.2012.04.001 22564542

[pei310062-bib-0008] Bittebière, A.‐K. , Vandenkoornhuyse, P. , Maluenda, E. , Gareil, A. , Dheilly, A. , Coudouel, S. , Bahin, M. , & Mony, C. (2019). Plant spatial structure of plant communities determines arbuscular mycorrhizal fungal community assembly. Journal of Ecology, 108, 546–560. 10.1111/1365-2745.13279

[pei310062-bib-0009] Borneman, J. , & Hartin, R. J. (2000). PCR primers that amplify fungal rRNA genes from environmental samples. Applied and Environmental Microbiology, 66, 4356–4460. 10.1128/AEM.66.10.4356-4360.2000 11010882 PMC92308

[pei310062-bib-0010] Bulgarelli, D. , Garrido‐Oter, R. , Münch, P. C. , Weiman, A. , Dröge, J. , Pan, Y. , McHardy, A. C. , & Schulze‐Lefert, P. (2015). Structure and function of the bacterial root microbiota in wild and domesticated barley. Cell Host Microbiology, 17, 392–403. 10.1016/j.chom.2015.01.011 PMC4362959 25732064

[pei310062-bib-0011] Chable, V. , Nuijten, E. , Costanzo, A. , Goldringer, I. , Bocci, R. , Oehen, B. , Rey, F. , Fasoula, D. , Feher, J. , Keskitalo, M. , Koller, B. , Omirou, M. , Mendes‐Moreira, P. , van Frank, G. , Naino Jika, A. K. , Thomas, M. , & Rossi, A. (2020). Embedding cultivated diversity in society for agro‐ecological transition. Sustainability, 12, 784. 10.3390/su12030784

[pei310062-bib-0012] Cheatham, M. R. , Rouse, M. N. , Esker, P. D. , Ignacio, S. , Pradel, W. , Raymundo, R. , Sparks, A. H. , Forbes, G. A. , Gordon, T. R. , & Garrett, K. A. (2009). Beyond yield: Plant disease in the context of ecosystem services. Phytopathology, 99, 1228–1236. 10.1094/PHYTO-99-11-1228b 19821726

[pei310062-bib-0013] Crous, P. W. , Gams, W. , Stalpers, J. A. , Robert, V. , & Stegehuis, G. (2004). MycoBank: An online initiative to launch mycology into the 21st century. Studies in Mycology, 50, 19–22.

[pei310062-bib-0014] De Cáceres, M. , Legendre, P. , & Moretti, M. (2010). Improving indicator species analysis by combining groups of sites. Oikos, 119, 1674–1684. 10.1111/j.1600-0706.2010.18334.x.

[pei310062-bib-0015] Dedryver, F. , Paillard, S. , Mallard, S. , Robert, O. , Trottet, M. , Nègre, S. , Verplancke, G. , & Jahier, J. (2009). Characterization of genetic components involved in durable resistance to stripe rust in the bread wheat ‘Renan’. Phytopathology, 99, 968–973. 10.1094/PHYTO-99-8-0968. 19594316

[pei310062-bib-0016] Denison, R. F. (2011). Past evolutionary tradeoffs represent opportunities for crop genetic improvement and increased human lifespan. Evolutionary Applications, 4, 216–224. 10.1111/j.1752-4571.2010.00158.x 25567969 PMC3352550

[pei310062-bib-0017] Döring, T. F. , Knapp, S. , Kovacs, G. , Murphy, K. , & Wolfe, M. S. (2011). Evolutionary plant breeding in cereals‐into a new era. Sustainability, 3, 1944–1971. 10.3390/su3101944

[pei310062-bib-0018] Dormann, C. F. , Elith, J. , Bacher, S. , Buchmann, C. , Carl, G. , Carré, G. , García Marquéz, J. R. , Gruber, B. , Lafourcade, B. , Leitão, P. J. , Münkemüller, T. , McClean, C. , Osborne, P. E. , Reineking, B. , Schröder, B. , Skidmore, A. K. , Zurell, D. , & Lautenbach, S. (2013). Collinearity: A review of methods to deal with it and a simulation study evaluating their performance. Ecography, 36, 27–46. 10.1111/j.1600-0587.2012.07348.x.

[pei310062-bib-0019] Duhamel, M. , & Vandenkoornhuyse, P. (2013). Sustainable agriculture: Possible trajectories from mutualistic symbiosis and plant neodomestication. Trends in Plant Science, 18, 597–600. 10.1016/j.tplants.2013.08.010 24055138

[pei310062-bib-0020] Escudié, F. , Auer, L. , Vernard, M. , Mariadassou, M. , Cauquil, L. , Vidal, K. , Maman, S. , Hernandez‐Raquet, G. , Combes, S. , & Pascal, G. (2018). FROGS: Find, rapidly, OTUS with Galaxy solution. Bioinformatics, 34, 1287–1294. 10.1093/bioinformatics/btx791 29228191

[pei310062-bib-0021] Fan, K. , Delgado‐Baquerizo, M. , Guo, X. , Wang, D. , Wu, Y. , Zhu, M. , Yu, W. , Yao, H. , Zhu, Y. , & Chu, H. (2019). Suppressed N fixation and diazotrophs after four decades of fertilization. Microbiome, 7, 143. 10.1186/s40168-019-0757-8 31672173 PMC6824023

[pei310062-bib-0022] FAO , Bélanger, J. , & Pilling, D. (2019). The State of the World’s Biodiversity for Food and Agriculture (Eds.). FAO Commission on Genetic Resources for Food and Agriculture Assessments, Rome, 572 pp.

[pei310062-bib-0023] Fox, J. , & Weisberg, S. (2019). An R companion to applied regression (3rd ed). Sage.

[pei310062-bib-0024] Gervais, L. , Dedryver, F. , Morlais, J. Y. , Bodusseau, V. , Nègre, S. , Bilous, M. , Groos, C. , & Trottet, M. (2003). Mapping of quantitative trait loci for field resistance to *Fusarium* head blight in an European winter wheat. Theoretical and Applied Genetics, 106, 961–970. 10.1007/s00122-002-1160-5. 12671743

[pei310062-bib-0025] Guo, J. , Ling, N. , Chen, Z. , Xue, C. , Li, L. , Liu, L. , Gao, L. , Wang, M. , Ruan, J. , Guo, S. , Vandenkoornhuyse, P. , & Shen, Q. (2020). Soil fungal assemblage complexity is dependent on soil fertility and dominated by deterministic processes. New Phytologist, 226, 232–243. 10.1111/nph.16345 31778576

[pei310062-bib-0026] Haudry, A. , Cenci, A. , Ravel, C. , Bataillon, T. , Brunel, D. , Poncet, C. , Hochu, I. , Poirier, S. , Santoni, S. , Glémin, S. , & David, J. (2007). Grinding up wheat: A massive loss of nucleotide diversity since domestication. Molecular Biology and Evolution, 24, 1506–1517. 10.1093/molbev/msm077 17443011

[pei310062-bib-0027] Hohmann, P. , & Messmer, M. (2017). Breeding for mycorrhizal symbiosis: Focus on disease resistance. Euphytica, 213, 1–11. 10.1007/s10681-017-1900-x

[pei310062-bib-0028] Iannucci, A. , Canfora, L. , Nigro, F. , De Vita, P. , & Beleggia, R. (2021). Relationships between root morphology, root exudate compounds and rhizosphere microbial community in durum wheat. Applied Soil Ecology, 158, 10.1016/j.apsoil.2020.103781. 103781

[pei310062-bib-0029] Kiers, E. T. , Duhamel, M. , Beesetty, Y. , Mensah, J. A. , Franken, O. , Verbruggen, E. , Felbaum, C. R. , Kowalchuk, G. A. , Hart, M. M. , Bago, A. , Palmer, T. M. , West, S. A. , Vandenkoornhuyse, P. , Jansa, J. , & Bücking, H. (2011). Reciprocal rewards stabilize cooperation in the mycorrhizal symbiosis. Science, 333, 880–882. 10.1126/science.1208473. 21836016

[pei310062-bib-0030] Kiers, E. T. , Hutton, M. G. , & Denison, F. (2007). Human selection and the relaxation of legume defences against ineffective rhizobia. Proceedings of the Royal Society B: Biological Science, 274, 3119–3126. https://doi.org/10.1098/rspb.2007.1187 PMC2293947 17939985

[pei310062-bib-0031] Kiers, E. T. , Rousseau, R. A. , West, S. A. , & Denison, R. F. (2003). Host sanctions and the legume–rhizobium mutualism. Nature, 425, 78–81. 10.1038/nature01931 12955144

[pei310062-bib-0032] Kozich, J. J. , Westcott, S. L. , Baxter, N. T. , Highlander, S. K. , & Schloss, P. D. (2013). Development of a dual‐index sequencing strategy and curation pipeline for analyzing amplicon sequence data on the MiSeq Illumina sequencing platform. Applied and Environmental Microbiology, 79, 5112–5120. 10.1128/AEM.01043-13 23793624 PMC3753973

[pei310062-bib-0033] Kuznetsova, K. , Brockhoff, P. B. , & Bojesen Christensen, R. H. (2017). lmerTest package: Tests in linear mixed effects models. Journal of Statistical Software, 82, 1548–7660.

[pei310062-bib-0034] Lammerts van Bueren, E. T. , Struik, P. C. , van Eekeren, N. , & Nuijten, E. (2018). Towards resilience through systems‐based plant breeding. A review. Agronomy and Sustainable Development, 5, 38–42. 10.1007/s13593-018-0522-6 PMC6417397 30956692

[pei310062-bib-0035] Martin‐Robles, N. , Lehmann, A. , Seco, E. , Aroca, R. , Rillig, M. C. , & Milla, R. (2018). Impacts of domestication on the arbuscular mycorrhizal symbiosis of 27 crop species. New Phytologist, 218, 322–334. 10.1111/nph.14962. 29281758

[pei310062-bib-0036] Marx, J. (2004). The roots of plant–microbe collaborations. Science, 304, 234–236. 10.1126/science.304.5668.234 15073365

[pei310062-bib-0038] Mony, C. , Gaudu, V. , Ricono, C. , Jambon, O. , & Vandenkoornhuyse, P. (2021). Plant neighbours shape fungal assemblages associated with plant roots: A new understanding of niche‐partitioning in plant communities. Functional Ecology, 35, 1768–1782. 10.1111/nph.16262.

[pei310062-bib-0039] Nguyen, N. H. , Song, Z. , Bates, S. T. , Branco, S. , Tedersoo, L. , Menke, J. , Schilling, J. S. , & Kennedy, P. G. (2016). FUNGuild: an open annotation tool for parsing fungal community datasets by ecological guild. Fungal Ecology, 20, 241–248. 10.1016/j.funeco.2015.06.006

[pei310062-bib-0040] Oksanen, J. (2019). Vegan: An introduction to ordination. http://cran.rproject.org/web/packages/vegan/vignettes/intro‐vegan.pdf

[pei310062-bib-0042] Pérez‐Jaramillo, J. E. , Carrión, V. J. , Bosse, M. , Ferrão, L. F. V. , de Hollander, M. , Garcia, A. A. F. , Ramírez, C. A. , Mendes, R. , & Raaijmakers, J. M. (2017). Linking rhizosphere microbiome composition of wild and domesticated *Phaseolus vulgaris* to genotypic and root phenotypic traits. The ISME Journal, 11, 2244–2257. 10.1038/ismej.2017.85 28585939 PMC5607367

[pei310062-bib-0043] Pérez‐Jaramillo, J. E. , Mendes, R. , & Raaijmakers, J. M. (2016). Impact of plant domestication on rhizosphere microbiome assembly and functions. Plant Molecular Biology, 90, 635–644. 10.1007/s11103-015-0337-7 26085172 PMC4819786

[pei310062-bib-0044] Pinheiro, J. , Bates, D. , DebRoy, S. , Sarkar, D. , & R Core Team (2020). nlme: Linear and nonlinear mixed effects models. R package version 3.1‐144.

[pei310062-bib-0045] Proctor, C. , & He, Y. H. (2017). Quantifying root extracts and exudates of sedge and shrub in relation to root morphology. Soil Biology and Biochemistry, 114, 168–180. 10.1016/j.soilbio.2017.07.006.

[pei310062-bib-0046] Quast, C. , Pruesse, E. , Yilmaz, P. , Gerken, J. , Schweer, T. , Yarza, P. , Peplies, J. , & Glockner, F. O. (2013). The SILVA ribosomal RNA gene database project: Improved data processing and web‐based tools. Nucleic Acids Research, 41, D590–D596. 10.1093/nar/gks1219. 23193283 PMC3531112

[pei310062-bib-0065] R Core Team (2016). R: A Language and Environment for Statistical Computing. Vienna, Austria: R Foundation for Statistical Computing. https://www.R‐project.org/

[pei310062-bib-0048] Roussel, V. , Leisova, L. , Exbrayat, F. , Stehno, Z. , & Balfourier, F. (2005). SSR allelic diversity changes in 480 European bread wheat varieties released from 1840 to 2000. Theoretical and Applied Genetics, 111, 162–170. 10.1007/s00122-005-2014-8 15887038

[pei310062-bib-0049] Saleem, M. , Law, A. D. , Radhi Sahib, M. , Pervaiz, Z. H. , & Zhang, Q. (2018). Impact of root system architecture on rhizosphere and root microbiome. Rhizosphere, 6, 47–51. 10.1016/j.rhisph.2018.02.003

[pei310062-bib-0050] Sasse, J. , Martinoia, E. , & Northern, T. (2018). Feed your friends: Do plant exudates shape the root microbiome ? Trends in Plant Science, 23, 25–45. 10.1016/j.tplants.2017.09.003 29050989

[pei310062-bib-0051] Tilman, D. , Balzer, C. , Hill, J. , & Befort, B. L. (2011). Global food demand and the sustainable intensification of agriculture. Proceedings of the National Academy of Sciences of the USA, 108, 20260–20264. 10.1073/pnas.1116437108 22106295 PMC3250154

[pei310062-bib-0052] Trivedi, P. , Leach, J. E. , Tringe, S. G. , Sa, T. , & Singh, B. K. (2020). Plant–microbiome interactions: From community assembly to plant health. Nature Reviews in Microbiology, 18, 607–621. 10.1038/s41579-020-0412-1 32788714

[pei310062-bib-0054] van de Wouw, M. , van Hintum, T. , Kik, C. , van Treuren, R. , & Visser, B. (2010). Genetic diversity trends in twentieth century crop cultivars: A meta‐analysis. Theoretical and Applied Genetics, 120, 1241–1252. 10.1007/s00122-009-1252-6 20054521 PMC2839474

[pei310062-bib-0055] Van Lê, A. , Quaiser, A. , Duhamel, M. , Michon‐Coudouel, S. , Dufresne, A. , & Vandenkoornhuyse, P. (2017). Ecophylogeney of the endospheric root fungal microbiome of co‐occurring *Agrostis stolonifera* . Peer Journal, 5, e3454. 10.7717/peerj.3454 PMC5466812 28607843

[pei310062-bib-0056] Vandenkoornhuyse, P. , Quaiser, A. , Duhamel, M. , Le Van, A. , & Dufresne, A. (2015). The importance of the microbiome of the plant holobiont. New Phytologist, 206, 1196–1206. 10.1111/nph.13312 25655016

[pei310062-bib-0053] Valente, J. , Gerin, F. , Le Gouis, J. , Moënne‐Loccoz, Y. , & Prigent‐Combaret, C. (2019). Ancient wheat varieties have a higher ability to interact with plant growth‐promoting rhizobacteria. Plant Cell and Environment, 43, 246–260. 10.1111/pce.13652 31509886

[pei310062-bib-0064] Valente, J , Gerin, F , Le Gouis, J , Moënne‐Loccoz, Y , & Prigent‐Combaret, C (2020). Ancient wheat varieties have a higher ability to interact with plant growth‐promoting rhizobacteria. Plant Cell & Environment, 43, 246–260. 10.1111/pce.13652 31509886

[pei310062-bib-0057] Vannier, N. , Agler, M. , & Hacquard, S. (2019). Microbiota‐mediated disease resistance in plants. PLoS Pathogens, 15, e1007740. 10.1371/2Fjournal.ppat.1007740 31194849 PMC6564022

[pei310062-bib-0058] Vannier, N. , Mony, C. , Bittebière, A.‐K. , Michon‐Coudouel, S. , Biget, M. , & Vandenkoornhuyse, P. (2018). A microorganims’ journey between plant generations. Microbiome, 6, 79. 10.1186/s40168-018-0459-7 29695286 PMC5918900

[pei310062-bib-0059] Verbruggen, E. , & Kiers, E. T. (2010). Evolutionary ecology of mycorrhizal functional diversity in agricultural systems. Evolutionary Applications, 3, 547–560. 10.1111/j.1752-4571.2010.00145.x 25567946 PMC3352509

[pei310062-bib-0060] Wei, Z. , & Jousset, A. (2017). Plant breeding goes microbial. Trends in Plant Science, 22, 555–558. 10.1016/j.tplants.2017.05.009 28592368

[pei310062-bib-0061] Wille, L. , Messmer, M. M. , Studer, B. , & Hohmann, P. (2019). Insights to plant‐microbe interactions provide opportunities to improve resistance breeding against root diseases in grain legumes. Plant Cell and Environment, 42, 20–40. 10.1111/pce.13214 29645277

[pei310062-bib-0062] Wuest, S. E. , Peter, R. , & Niklaus, P. A. (2021). Ecological and evolutionary approaches to improving crop variety mixtures. Nature Ecology and Evolution, 5, 1068–1077. 10.1038/s41559-021-01497-x 34211140

[pei310062-bib-0063] Xiong, C. , Zhu, Y.‐G. , Wang, J.‐T. , Singh, B. , Han, L.‐L. , Shen, J.‐P. , Li, P.‐P. , Wang, G.‐B. , Wu, C.‐F. , Ge, A.‐H. , Zhang, L.‐M. , & He, J.‐Z. (2021). Host selection shapes crop microbiome assembly and network complexity. New Phytologist, 229, 1091–1104. 10.1111/nph.16890 32852792

