# Supplementary material for: Differentiation of endospheric microbiota in ancient and modern wheat cultivar roots
Source: Plant Environ Interact. 2021 Oct 19;2(5):235–48. doi: 10.1002/pei3.10062 (PMC10168034; doi:10.1002/pei3.10062)
Supplement: Supplementary file 1 — Supplementary Material [file PEI3-2-235-s001.docx]

**Differentiation of endospheric microbiota in ancient and**

**modern wheat cultivars roots**

**Mauger S^1*^, Ricono C^1*^, Mony C^1^, Chable V^2^, Serpolay E^2^, Biget M^1^, Vandenkoornhuyse P^1^**

*******

**Supplementary material**

*******

**Supplementary material**

**Pathogens**

Ten pathogen sequence-clusters were identified in fungi (1 Helicobasidium (Pucciniomycetes); three Magnaporthaceae (Sordariomycetes); three Microascales, including one Graphium penicillioides (Sordariomycetes); two Leptosphaeria maculans “lepidii” group (Sprague et al. 2007) and one Parastagonospora nodorum (Chooi et al. 2014) (Dothideomycetes)).

A total of 87 pathogen sequence-clusters were identified among bacteria (11 Streptomycetaceae (Streptomyces) (Zhang et al. 2018); 3 Clostridiaceae; eight Enterobacteriaceae including one Enterobacter, two Erwinia, two Pantoea and three Serratia (Rosa et al. 2019); 27 Sphingomonadaceae (Sphingomonas) (Bachabi et al. 2018); six Burkholderiaceae including four Acidovorax and 2 Burkholderia (Di Cenzo et al. 2019); 32 Pseudomonadaceae (Pseudomonas) (Licciardello et al. 2017)).

**Figure and Tables :**

Fungi


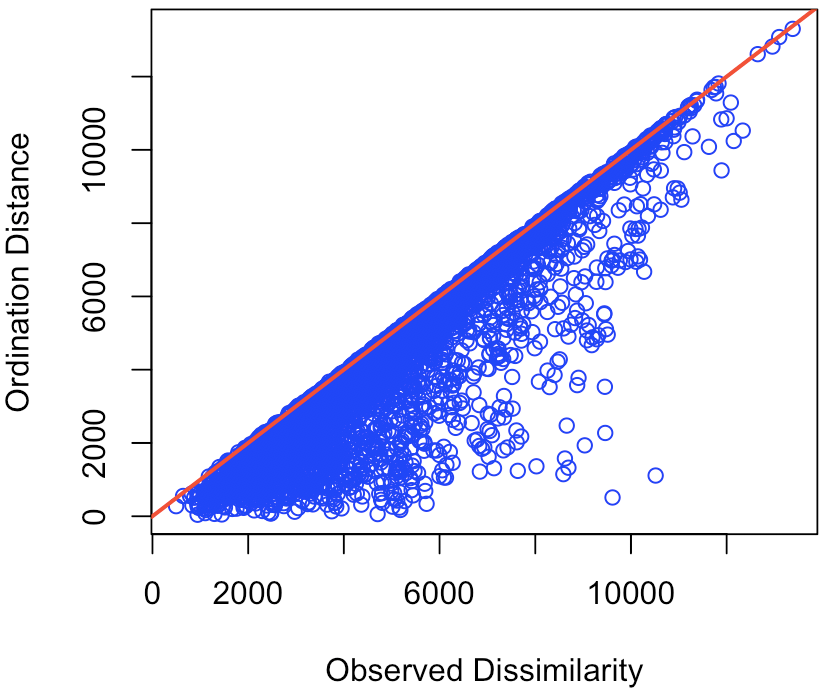

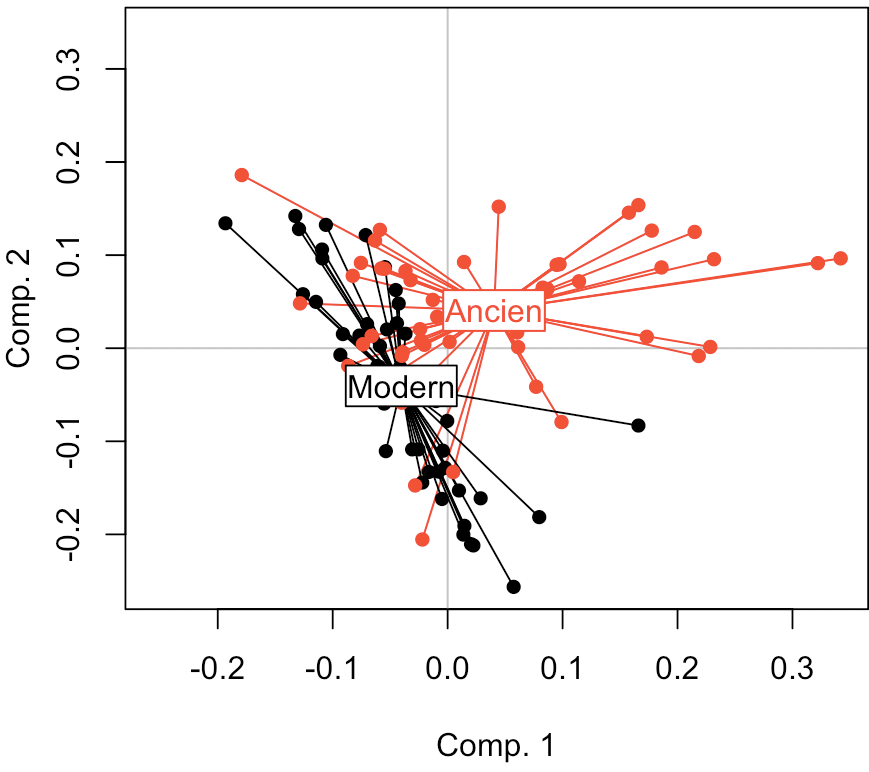


Bacteria


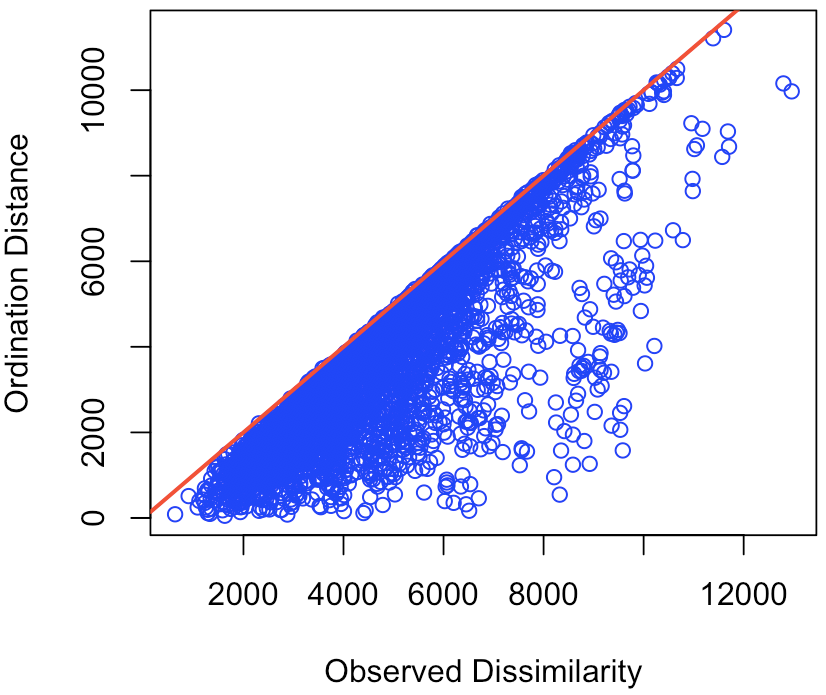

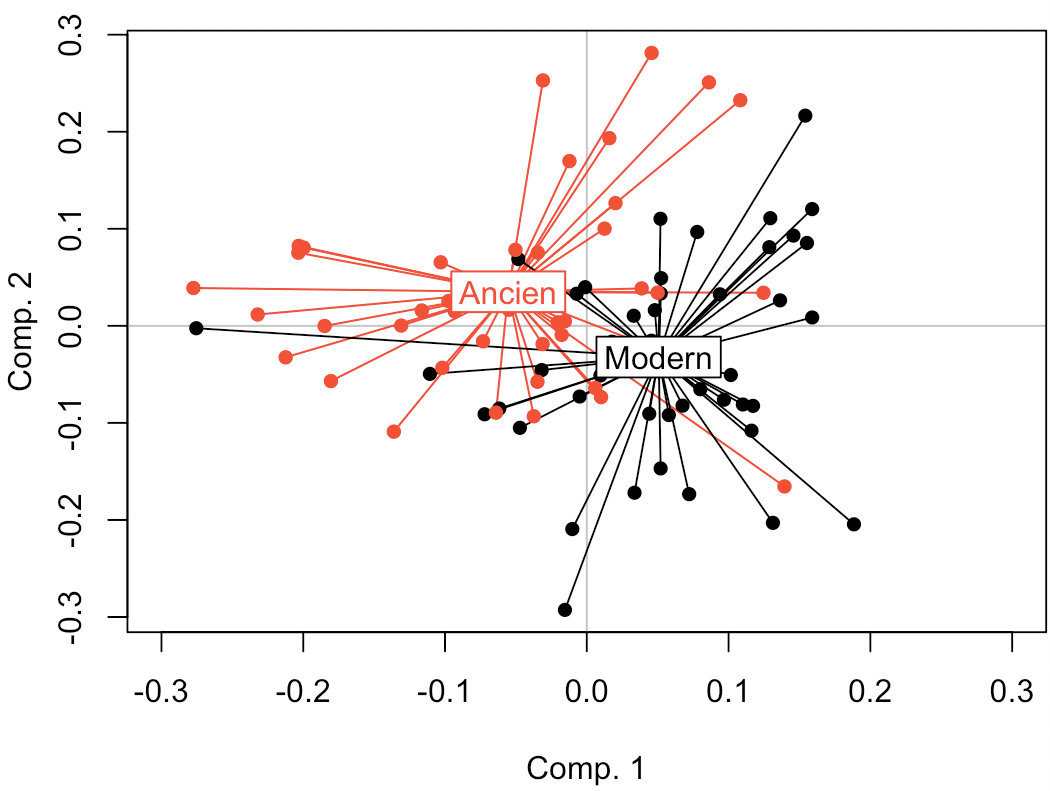


**Figure S1:** PCoA analyses of fungal and bacterial root-endospheric microbiota in modern and ancient wheat cultivars.

**Table S1** Partial Least Squares Discriminant Analysis (PLS-DA) of the bacterial and fungal community in ancient (A) and modern (M) cultivars tested on the main phyla

| Group | P | Mean classification error |
| --- | --- | --- |
| All bacteria | 0.001 | 11 |
| Proteobacteria | 0.001 | 16.9 |
| Bacteroidetes | 0.001 | 10.4 |
| Actinobacteria | NS | - |
| Firmicutes | 0.024 | 21.8 |
| Acidobacteria | 0.027 | 39.1 |
|  |  |  |
| All fungi | 0.001 | 23.6 |
| Ascomycetes | 0.001 | 23.2 |
| Basidiomycetes | 0.002 | 26.4 |
| Other fungi | 0.034 | 37.6 |

**Table S2 :** Results of Permutational Multivariate Analysis of Variance (Adonis) comparing the root-microbiota endosphere in ancient vs modern wheat cultivars

|  | R^2^ | P |
| --- | --- | --- |
| Bacteria | 0.15 | *** |
| Fungi | 0.13 | *** |

**Table S3 :** Synthetized results of the ‘indicator species’ of modern or ancient wheat cultivar category. The numbers in the table corresponded to the number of sequence-clusters found by permutation tests statistically significantly higher than expected in a category compared to the other.

|  | Modern cultivars | Ancient cultivars |
| --- | --- | --- |
| **Bacteria** |  |  |
| Acidobacteria | 0 | 7 |
| Actinobacteria | 4 | 21 |
| Alphaproteobacteria | 13 | 84 |
| Bacteroidetes | 59 | 68 |
| Deltaproteobacteria | 10 | 16 |
| Fibrobacteres | 2 | 0 |
| Firmicutes | 11 | 12 |
| Gammaproteobacteria | 65 | 38 |
| Gemmatimonadetes | 0 | 4 |
| Nitrospirae | 0 | 1 |
| Spirochaetes | 3 | 0 |
| Unknown affiliation | 1 | 5 |
|  |  |  |
| **Fungi** |  |  |
| Ascomycota | 17 | 6 |
| Basidiomycota | 4 | 7 |
| Chytridiomycetes | 1 | 0 |
| Glomeromycota | 4 | 0 |
| Unknown affiliation | 3 | 3 |

**Table S3 :** Detailed results of the ‘indicator species’ analyses of modern or ancient wheat cultivar category.

| #Cluster | stat | p.value | level of significance | |  |  |  |  |  |
| --- | --- | --- | --- | --- | --- | --- | --- | --- | --- |
| **Fungi - Ancient Cultivars** | |  |  |  |  |  |  |  |  |
| Cluster_124 | 0.278 | 0.0004 | *** | Ascomycota | Dothideomycetes | Pleosporales | Leptosphaeriaceae | Leptosphaeria | Leptosphaeria maculans 'lepidii' group |
| Cluster_152 | 0.353 | 0.0004 | *** | Ascomycota | Dothideomycetes | Pleosporales | unknown family | unknown genus | fungal sp. |
| Cluster_99 | 0.387 | 0.0001 | *** | Ascomycota | Dothideomycetes | Pleosporales | unknown family | unknown genus | Phoma sp. |
| Cluster_3 | 0.535 | 0.0001 | *** | Ascomycota | Multi-affiliation | Multi-affiliation | Multi-affiliation | Multi-affiliation | Multi-affiliation |
| Cluster_717 | 0.239 | 0.0147 | * | Ascomycota | Sordariomycetes | Multi-affiliation | Multi-affiliation | Multi-affiliation | Multi-affiliation |
| Cluster_26 | 0.45 | 0.0001 | *** | Ascomycota | Taphrinomycetes | Taphrinales | Multi-affiliation | Multi-affiliation | Multi-affiliation |
| Cluster_57 | 0.222 | 0.0143 | * | Basidiomycota | Agaricostilbomycetes | Agaricostilbales | Agaricostilbaceae | Bensingtonia | Kondoa changbaiensis |
| Cluster_24 | 0.4 | 0.0001 | *** | Basidiomycota | Multi-affiliation | Multi-affiliation | Multi-affiliation | Multi-affiliation | Multi-affiliation |
| Cluster_79 | 0.133 | 0.0009 | *** | Basidiomycota | Multi-affiliation | Multi-affiliation | Multi-affiliation | Multi-affiliation | Multi-affiliation |
| Cluster_14 | 0.298 | 0.0017 | ** | Basidiomycota | Tremellomycetes | Cystofilobasidiales | Cystofilobasidiaceae | Multi-affiliation | Multi-affiliation |
| Cluster_940 | 0.268 | 0.0161 | * | Basidiomycota | Tremellomycetes | Cystofilobasidiales | Cystofilobasidiaceae | Multi-affiliation | Multi-affiliation |
| Cluster_488 | 0.262 | 0.0148 | * | Basidiomycota | Tremellomycetes | Multi-affiliation | Multi-affiliation | Multi-affiliation | Multi-affiliation |
| Cluster_230 | 0.326 | 0.0014 | ** | Basidiomycota | Tremellomycetes | Tremellales | Multi-affiliation | Multi-affiliation | Multi-affiliation |
| Cluster_104 | 0.208 | 0.0356 | * | Multi-affiliation | Multi-affiliation | Multi-affiliation | Multi-affiliation | Multi-affiliation | Multi-affiliation |
| Cluster_68 | 0.23 | 0.0261 | * | Multi-affiliation | Multi-affiliation | Multi-affiliation | Multi-affiliation | Multi-affiliation | Multi-affiliation |
| Cluster_8 | 0.401 | 0.0001 | *** | Multi-affiliation | Multi-affiliation | Multi-affiliation | Multi-affiliation | Multi-affiliation | Multi-affiliation |
|  |  |  |  |  |  |  |  |  |  |
| **Fungi - Modern Cultivars** | |  |  |  |  |  |  |  |  |
| Cluster_163 | 0.263 | 0.0093 | ** | Ascomycota | Multi-affiliation | Multi-affiliation | Multi-affiliation | Multi-affiliation | Multi-affiliation |
| Cluster_7 | 0.442 | 0.0001 | *** | Ascomycota | Multi-affiliation | Multi-affiliation | Multi-affiliation | Multi-affiliation | Multi-affiliation |
| Cluster_9 | 0.501 | 0.0001 | *** | Ascomycota | Multi-affiliation | Multi-affiliation | Multi-affiliation | Multi-affiliation | Multi-affiliation |
| Cluster_20 | 0.439 | 0.0001 | *** | Ascomycota | Orbiliomycetes | Orbiliales | Orbiliaceae | Multi-affiliation | Multi-affiliation |
| Cluster_73 | 0.443 | 0.0001 | *** | Ascomycota | Pezizomycetes | Pezizales | Multi-affiliation | Multi-affiliation | Multi-affiliation |
| Cluster_30 | 0.323 | 0.001 | *** | Ascomycota | Saccharomycetes | Saccharomycetales | unknown family | Candida | Candida sorboxylosa |
| Cluster_435 | 0.311 | 0.002 | ** | Ascomycota | Saccharomycetes | Saccharomycetales | unknown family | Multi-affiliation | Multi-affiliation |
| Cluster_293 | 0.226 | 0.0036 | ** | Ascomycota | Sordariomycetes | Magnaporthales | Magnaporthaceae | Multi-affiliation | Multi-affiliation |
| Cluster_32 | 0.206 | 0.0071 | ** | Ascomycota | Sordariomycetes | Magnaporthales | Magnaporthaceae | Multi-affiliation | Multi-affiliation |
| Cluster_49 | 0.217 | 0.0112 | * | Ascomycota | Sordariomycetes | Microascales | Multi-affiliation | Multi-affiliation | Multi-affiliation |
| Cluster_271 | 0.373 | 0.0001 | *** | Ascomycota | Sordariomycetes | Multi-affiliation | Multi-affiliation | Multi-affiliation | Multi-affiliation |
| Cluster_36 | 0.247 | 0.0061 | ** | Ascomycota | Sordariomycetes | Multi-affiliation | Multi-affiliation | Multi-affiliation | Multi-affiliation |
| Cluster_44 | 0.456 | 0.0001 | *** | Ascomycota | Sordariomycetes | Multi-affiliation | Multi-affiliation | Multi-affiliation | Multi-affiliation |
| Cluster_529 | 0.284 | 0.0028 | ** | Ascomycota | Sordariomycetes | Multi-affiliation | Multi-affiliation | Multi-affiliation | Multi-affiliation |
| Cluster_56 | 0.326 | 0.0015 | ** | Ascomycota | Sordariomycetes | Multi-affiliation | Multi-affiliation | Multi-affiliation | Multi-affiliation |
| Cluster_6 | 0.287 | 0.0045 | ** | Ascomycota | Sordariomycetes | Multi-affiliation | Multi-affiliation | Multi-affiliation | Multi-affiliation |
| Cluster_18 | 0.427 | 0.0001 | *** | Ascomycota | Sordariomycetes | Sordariales | Multi-affiliation | Multi-affiliation | Multi-affiliation |
| Cluster_211 | 0.308 | 0.0007 | *** | Basidiomycota | Multi-affiliation | Multi-affiliation | Multi-affiliation | Multi-affiliation | Multi-affiliation |
| Cluster_33 | 0.426 | 0.0001 | *** | Basidiomycota | Multi-affiliation | Multi-affiliation | Multi-affiliation | Multi-affiliation | Multi-affiliation |
| Cluster_333 | 0.207 | 0.0294 | * | Basidiomycota | Multi-affiliation | Multi-affiliation | Multi-affiliation | Multi-affiliation | Multi-affiliation |
| Cluster_5 | 0.407 | 0.0001 | *** | Basidiomycota | Multi-affiliation | Multi-affiliation | Multi-affiliation | Multi-affiliation | Multi-affiliation |
| Cluster_256 | 0.213 | 0.0323 | * | Chytridiomycota | Chytridiomycetes | Lobulomycetales | Lobulomycetaceae | Multi-affiliation | Multi-affiliation |
| Cluster_119 | 0.39 | 0.0001 | *** | Glomeromycota | Glomeromycetes | Glomerales | Multi-affiliation | Multi-affiliation | Multi-affiliation |
| Cluster_29 | 0.378 | 0.0002 | *** | Glomeromycota | Glomeromycetes | Glomerales | Multi-affiliation | Multi-affiliation | Multi-affiliation |
| Cluster_1739 | 0.224 | 0.0261 | * | Glomeromycota | Glomeromycetes | Glomerales | unknown family | unknown genus | unknown species |
| Cluster_200 | 0.366 | 0.0001 | *** | Glomeromycota | Glomeromycetes | Glomerales | unknown family | unknown genus | unknown species |
| Cluster_17 | 0.311 | 0.0014 | ** | Multi-affiliation | Multi-affiliation | Multi-affiliation | Multi-affiliation | Multi-affiliation | Multi-affiliation |
| Cluster_178 | 0.224 | 0.0289 | * | unknown phylum | unknown class | Mortierellales | Mortierellaceae | Mortierella | Mortierella parvispora |
| Cluster_426 | 0.275 | 0.0066 | ** | unknown phylum | unknown class | Mortierellales | Multi-affiliation | Multi-affiliation | Multi-affiliation |
|  |  |  |  |  |  |  |  |  |  |
| **Bacteria - Ancient Cultivars** | | |  |  |  |  |  |  |  |
| Cluster_822 | 0.269 | 0.0087 | ** | Acidobacteria | Acidobacteriia | Solibacterales | Solibacteraceae (Subgroup 3) | Bryobacter | unknown species |
| Cluster_1228 | 0.208 | 0.0368 | * | Acidobacteria | Holophagae | Subgroup 7 | unknown family | unknown genus | unknown species |
| Cluster_982 | 0.188 | 0.0488 | * | Acidobacteria | Subgroup 25 | unknown order | unknown family | unknown genus | unknown species |
| Cluster_365 | 0.329 | 0.0002 | *** | Acidobacteria | Thermoanaerobaculia | Thermoanaerobaculales | Thermoanaerobaculaceae | Subgroup 10 | Multi-affiliation |
| Cluster_626 | 0.248 | 0.0201 | * | Acidobacteria | Thermoanaerobaculia | Thermoanaerobaculales | Thermoanaerobaculaceae | Subgroup 10 | unknown species |
| Cluster_829 | 0.301 | 0.0011 | ** | Acidobacteria | Thermoanaerobaculia | Thermoanaerobaculales | Thermoanaerobaculaceae | Subgroup 10 | unknown species |
| Cluster_1714 | 0.209 | 0.0396 | * | Acidobacteria | Thermoanaerobaculia | Thermoanaerobaculales | Thermoanaerobaculaceae | Subgroup 10 | unknown species |
| Cluster_263 | 0.231 | 0.0244 | * | Actinobacteria | Actinobacteria | Streptomycetales | Streptomycetaceae | Streptomyces | Multi-affiliation |
| Cluster_341 | 0.389 | 0.0001 | *** | Actinobacteria | Thermoleophilia | Gaiellales | Gaiellaceae | Gaiella | unknown species |
| Cluster_355 | 0.213 | 0.0395 | * | Actinobacteria | Thermoleophilia | Gaiellales | Gaiellaceae | Gaiella | unknown species |
| Cluster_542 | 0.297 | 0.0037 | ** | Actinobacteria | Thermoleophilia | Gaiellales | Gaiellaceae | Gaiella | unknown species |
| Cluster_815 | 0.257 | 0.0133 | * | Actinobacteria | Thermoleophilia | Gaiellales | Gaiellaceae | Gaiella | unknown species |
| Cluster_983 | 0.27 | 0.0015 | ** | Actinobacteria | Thermoleophilia | Gaiellales | Gaiellaceae | Gaiella | unknown species |
| Cluster_1148 | 0.239 | 0.0196 | * | Actinobacteria | Thermoleophilia | Gaiellales | Gaiellaceae | Gaiella | unknown species |
| Cluster_465 | 0.398 | 0.0001 | *** | Actinobacteria | Thermoleophilia | Gaiellales | unknown family | unknown genus | unknown species |
| Cluster_637 | 0.372 | 0.0002 | *** | Actinobacteria | Thermoleophilia | Gaiellales | unknown family | unknown genus | unknown species |
| Cluster_897 | 0.218 | 0.0278 | * | Actinobacteria | Thermoleophilia | Gaiellales | unknown family | unknown genus | unknown species |
| Cluster_1103 | 0.206 | 0.0464 | * | Actinobacteria | Thermoleophilia | Gaiellales | unknown family | unknown genus | unknown species |
| Cluster_1128 | 0.349 | 0.0002 | *** | Actinobacteria | Thermoleophilia | Gaiellales | unknown family | unknown genus | unknown species |
| Cluster_1737 | 0.214 | 0.039 | * | Actinobacteria | Thermoleophilia | Gaiellales | unknown family | unknown genus | unknown species |
| Cluster_1886 | 0.254 | 0.0053 | ** | Actinobacteria | Thermoleophilia | Gaiellales | unknown family | unknown genus | unknown species |
| Cluster_460 | 0.326 | 0.0014 | ** | Actinobacteria | Thermoleophilia | Solirubrobacterales | 67-14 | unknown genus | unknown species |
| Cluster_500 | 0.379 | 0.0001 | *** | Actinobacteria | Thermoleophilia | Solirubrobacterales | 67-14 | unknown genus | unknown species |
| Cluster_789 | 0.287 | 0.0015 | ** | Actinobacteria | Thermoleophilia | Solirubrobacterales | 67-14 | unknown genus | unknown species |
| Cluster_950 | 0.435 | 0.0001 | *** | Actinobacteria | Thermoleophilia | Solirubrobacterales | 67-14 | unknown genus | unknown species |
| Cluster_1212 | 0.277 | 0.0042 | ** | Actinobacteria | Thermoleophilia | Solirubrobacterales | 67-14 | unknown genus | unknown species |
| Cluster_1796 | 0.254 | 0.0115 | * | Actinobacteria | Thermoleophilia | Solirubrobacterales | 67-14 | unknown genus | unknown species |
| Cluster_552 | 0.359 | 0.0002 | *** | Actinobacteria | Thermoleophilia | Solirubrobacterales | Solirubrobacteraceae | Solirubrobacter | Multi-affiliation |
| Cluster_139 | 0.339 | 0.0011 | ** | Bacteria | Bacteroidetes | Bacteroidia | Cytophagales | Microscillaceae | unknown genus |
| Cluster_579 | 0.259 | 0.0118 | * | Bacteroidetes | Bacteroidia | Bacteroidetes VC2.1 Bac22 | unknown family | unknown genus | metagenome |
| Cluster_704 | 0.245 | 0.0121 | * | Bacteroidetes | Bacteroidia | Chitinophagales | Chitinophagaceae | Chitinophaga | Multi-affiliation |
| Cluster_498 | 0.218 | 0.0407 | * | Bacteroidetes | Bacteroidia | Chitinophagales | Chitinophagaceae | Dinghuibacter | unknown species |
| Cluster_888 | 0.25 | 0.0161 | * | Bacteroidetes | Bacteroidia | Chitinophagales | Chitinophagaceae | Dinghuibacter | unknown species |
| Cluster_40 | 0.438 | 0.0001 | *** | Bacteroidetes | Bacteroidia | Chitinophagales | Chitinophagaceae | Ferruginibacter | Ferruginibacter paludis |
| Cluster_54 | 0.322 | 0.0015 | ** | Bacteroidetes | Bacteroidia | Chitinophagales | Chitinophagaceae | Ferruginibacter | Multi-affiliation |
| Cluster_83 | 0.382 | 0.0003 | *** | Bacteroidetes | Bacteroidia | Chitinophagales | Chitinophagaceae | Ferruginibacter | Multi-affiliation |
| Cluster_100 | 0.345 | 0.0007 | *** | Bacteroidetes | Bacteroidia | Chitinophagales | Chitinophagaceae | Ferruginibacter | Multi-affiliation |
| Cluster_56 | 0.426 | 0.0001 | *** | Bacteroidetes | Bacteroidia | Chitinophagales | Chitinophagaceae | Ferruginibacter | unknown species |
| Cluster_66 | 0.439 | 0.0001 | *** | Bacteroidetes | Bacteroidia | Chitinophagales | Chitinophagaceae | Ferruginibacter | unknown species |
| Cluster_73 | 0.286 | 0.0058 | ** | Bacteroidetes | Bacteroidia | Chitinophagales | Chitinophagaceae | Ferruginibacter | unknown species |
| Cluster_101 | 0.342 | 0.001 | *** | Bacteroidetes | Bacteroidia | Chitinophagales | Chitinophagaceae | Ferruginibacter | unknown species |
| Cluster_161 | 0.219 | 0.001 | *** | Bacteroidetes | Bacteroidia | Chitinophagales | Chitinophagaceae | Ferruginibacter | unknown species |
| Cluster_211 | 0.421 | 0.0001 | *** | Bacteroidetes | Bacteroidia | Chitinophagales | Chitinophagaceae | Ferruginibacter | unknown species |
| Cluster_253 | 0.237 | 0.0145 | * | Bacteroidetes | Bacteroidia | Chitinophagales | Chitinophagaceae | Ferruginibacter | unknown species |
| Cluster_347 | 0.347 | 0.0008 | *** | Bacteroidetes | Bacteroidia | Chitinophagales | Chitinophagaceae | Ferruginibacter | unknown species |
| Cluster_371 | 0.291 | 0.0046 | ** | Bacteroidetes | Bacteroidia | Chitinophagales | Chitinophagaceae | Ferruginibacter | unknown species |
| Cluster_851 | 0.334 | 0.0001 | *** | Bacteroidetes | Bacteroidia | Chitinophagales | Chitinophagaceae | Ferruginibacter | unknown species |
| Cluster_860 | 0.268 | 0.0036 | ** | Bacteroidetes | Bacteroidia | Chitinophagales | Chitinophagaceae | Ferruginibacter | unknown species |
| Cluster_1052 | 0.235 | 0.0313 | * | Bacteroidetes | Bacteroidia | Chitinophagales | Chitinophagaceae | Ferruginibacter | unknown species |
| Cluster_1238 | 0.244 | 0.023 | * | Bacteroidetes | Bacteroidia | Chitinophagales | Chitinophagaceae | Ferruginibacter | unknown species |
| Cluster_1413 | 0.275 | 0.001 | *** | Bacteroidetes | Bacteroidia | Chitinophagales | Chitinophagaceae | Ferruginibacter | unknown species |
| Cluster_551 | 0.216 | 0.0391 | * | Bacteroidetes | Bacteroidia | Chitinophagales | Chitinophagaceae | Flavisolibacter | Multi-affiliation |
| Cluster_1235 | 0.24 | 0.014 | * | Bacteroidetes | Bacteroidia | Chitinophagales | Chitinophagaceae | Flavisolibacter | Multi-affiliation |
| Cluster_663 | 0.227 | 0.0246 | * | Bacteroidetes | Bacteroidia | Chitinophagales | Chitinophagaceae | Flavisolibacter | unknown species |
| Cluster_730 | 0.265 | 0.0088 | ** | Bacteroidetes | Bacteroidia | Chitinophagales | Chitinophagaceae | Flavisolibacter | unknown species |
| Cluster_168 | 0.467 | 0.0001 | *** | Bacteroidetes | Bacteroidia | Chitinophagales | Chitinophagaceae | Multi-affiliation | Multi-affiliation |
| Cluster_845 | 0.249 | 0.0194 | * | Bacteroidetes | Bacteroidia | Chitinophagales | Chitinophagaceae | Parafilimonas | unknown species |
| Cluster_154 | 0.322 | 0.0011 | ** | Bacteroidetes | Bacteroidia | Chitinophagales | Chitinophagaceae | Pseudoflavitalea | Multi-affiliation |
| Cluster_890 | 0.305 | 0.0028 | ** | Bacteroidetes | Bacteroidia | Chitinophagales | Chitinophagaceae | Sediminibacterium | Hypsibius dujardini |
| Cluster_978 | 0.373 | 0.0001 | *** | Bacteroidetes | Bacteroidia | Chitinophagales | Chitinophagaceae | Sediminibacterium | Multi-affiliation |
| Cluster_713 | 0.405 | 0.0001 | *** | Bacteroidetes | Bacteroidia | Chitinophagales | Chitinophagaceae | Sediminibacterium | unknown species |
| Cluster_768 | 0.26 | 0.0042 | ** | Bacteroidetes | Bacteroidia | Chitinophagales | Chitinophagaceae | Terrimonas | Terrimonas arctica |
| Cluster_173 | 0.292 | 0.0049 | ** | Bacteroidetes | Bacteroidia | Chitinophagales | Chitinophagaceae | Terrimonas | unknown species |
| Cluster_187 | 0.367 | 0.0001 | *** | Bacteroidetes | Bacteroidia | Chitinophagales | Chitinophagaceae | Terrimonas | unknown species |
| Cluster_340 | 0.299 | 0.0042 | ** | Bacteroidetes | Bacteroidia | Chitinophagales | Chitinophagaceae | Terrimonas | unknown species |
| Cluster_289 | 0.355 | 0.0005 | *** | Bacteroidetes | Bacteroidia | Chitinophagales | Chitinophagaceae | unknown genus | metagenome |
| Cluster_80 | 0.279 | 0.0075 | ** | Bacteroidetes | Bacteroidia | Chitinophagales | Chitinophagaceae | unknown genus | unknown species |
| Cluster_160 | 0.277 | 0.0054 | ** | Bacteroidetes | Bacteroidia | Chitinophagales | Chitinophagaceae | unknown genus | unknown species |
| Cluster_186 | 0.397 | 0.0003 | *** | Bacteroidetes | Bacteroidia | Chitinophagales | Chitinophagaceae | unknown genus | unknown species |
| Cluster_206 | 0.334 | 0.0015 | ** | Bacteroidetes | Bacteroidia | Chitinophagales | Chitinophagaceae | unknown genus | unknown species |
| Cluster_302 | 0.297 | 0.0047 | ** | Bacteroidetes | Bacteroidia | Chitinophagales | Chitinophagaceae | unknown genus | unknown species |
| Cluster_316 | 0.242 | 0.0209 | * | Bacteroidetes | Bacteroidia | Chitinophagales | Chitinophagaceae | unknown genus | unknown species |
| Cluster_356 | 0.234 | 0.0224 | * | Bacteroidetes | Bacteroidia | Chitinophagales | Chitinophagaceae | unknown genus | unknown species |
| Cluster_375 | 0.23 | 0.0283 | * | Bacteroidetes | Bacteroidia | Chitinophagales | Chitinophagaceae | unknown genus | unknown species |
| Cluster_678 | 0.302 | 0.0023 | ** | Bacteroidetes | Bacteroidia | Chitinophagales | Chitinophagaceae | unknown genus | unknown species |
| Cluster_1069 | 0.339 | 0.0004 | *** | Bacteroidetes | Bacteroidia | Chitinophagales | Chitinophagaceae | unknown genus | unknown species |
| Cluster_1379 | 0.267 | 0.0057 | ** | Bacteroidetes | Bacteroidia | Chitinophagales | Chitinophagaceae | unknown genus | unknown species |
| Cluster_1629 | 0.269 | 0.0109 | * | Bacteroidetes | Bacteroidia | Chitinophagales | Chitinophagaceae | unknown genus | unknown species |
| Cluster_19 | 0.561 | 0.0001 | *** | Bacteroidetes | Bacteroidia | Cytophagales | Amoebophilaceae | Candidatus Cardinium | Multi-affiliation |
| Cluster_71 | 0.42 | 0.0001 | *** | Bacteroidetes | Bacteroidia | Cytophagales | Microscillaceae | Chryseolinea | Multi-affiliation |
| Cluster_235 | 0.387 | 0.0001 | *** | Bacteroidetes | Bacteroidia | Cytophagales | Microscillaceae | Chryseolinea | unknown species |
| Cluster_535 | 0.292 | 0.0031 | ** | Bacteroidetes | Bacteroidia | Cytophagales | Microscillaceae | Ohtaekwangia | Bacteroidetes bacterium GS |
| Cluster_132 | 0.217 | 0.0397 | * | Bacteroidetes | Bacteroidia | Cytophagales | Microscillaceae | Ohtaekwangia | metagenome |
| Cluster_594 | 0.294 | 0.0046 | ** | Bacteroidetes | Bacteroidia | Cytophagales | Microscillaceae | Ohtaekwangia | metagenome |
| Cluster_765 | 0.21 | 0.0441 | * | Bacteroidetes | Bacteroidia | Cytophagales | Microscillaceae | Ohtaekwangia | unknown species |
| Cluster_138 | 0.305 | 0.002 | ** | Bacteroidetes | Bacteroidia | Cytophagales | Microscillaceae | unknown genus | metagenome |
| Cluster_36 | 0.333 | 0.0011 | ** | Bacteroidetes | Bacteroidia | Cytophagales | Microscillaceae | unknown genus | unknown species |
| Cluster_298 | 0.3 | 0.0042 | ** | Bacteroidetes | Bacteroidia | Cytophagales | Microscillaceae | unknown genus | unknown species |
| Cluster_361 | 0.461 | 0.0001 | *** | Bacteroidetes | Bacteroidia | Cytophagales | Microscillaceae | unknown genus | unknown species |
| Cluster_366 | 0.252 | 0.0156 | * | Bacteroidetes | Bacteroidia | Cytophagales | Microscillaceae | unknown genus | unknown species |
| Cluster_857 | 0.238 | 0.0151 | * | Bacteroidetes | Bacteroidia | Cytophagales | Microscillaceae | unknown genus | unknown species |
| Cluster_1 | 0.5 | 0.0001 | *** | Bacteroidetes | Bacteroidia | Flavobacteriales | Flavobacteriaceae | Flavobacterium | Multi-affiliation |
| Cluster_222 | 0.224 | 0.0343 | * | Bacteroidetes | Bacteroidia | Sphingobacteriales | Sphingobacteriaceae | Mucilaginibacter | Multi-affiliation |
| Cluster_545 | 0.275 | 0.0051 | ** | Bacteroidetes | Bacteroidia | Sphingobacteriales | Sphingobacteriaceae | Mucilaginibacter | unknown species |
| Cluster_47 | 0.296 | 0.0015 | ** | Bacteroidetes | Bacteroidia | Sphingobacteriales | Sphingobacteriaceae | Pedobacter | Multi-affiliation |
| Cluster_401 | 0.284 | 0.0042 | ** | Bacteroidetes | Bacteroidia | Sphingobacteriales | Sphingobacteriaceae | unknown genus | unknown species |
| Cluster_117 | 0.265 | 0.0052 | ** | Firmicutes | Bacilli | Bacillales | Bacillaceae | Bacillus | Multi-affiliation |
| Cluster_280 | 0.216 | 0.0377 | * | Firmicutes | Bacilli | Bacillales | Bacillaceae | Bacillus | Multi-affiliation |
| Cluster_424 | 0.247 | 0.0126 | * | Firmicutes | Bacilli | Bacillales | Bacillaceae | Bacillus | Multi-affiliation |
| Cluster_353 | 0.306 | 0.0034 | ** | Firmicutes | Bacilli | Bacillales | Bacillaceae | Bacillus | unknown species |
| Cluster_830 | 0.227 | 0.0191 | * | Firmicutes | Bacilli | Bacillales | Multi-affiliation | Multi-affiliation | Multi-affiliation |
| Cluster_164 | 0.235 | 0.0211 | * | Firmicutes | Bacilli | Bacillales | Paenibacillaceae | Paenibacillus | Multi-affiliation |
| Cluster_216 | 0.244 | 0.0201 | * | Firmicutes | Bacilli | Bacillales | Paenibacillaceae | Paenibacillus | Multi-affiliation |
| Cluster_1083 | 0.335 | 0.0011 | ** | Firmicutes | Bacilli | Bacillales | Planococcaceae | Sporosarcina | Multi-affiliation |
| Cluster_1079 | 0.248 | 0.0112 | * | Firmicutes | Bacilli | Bacillales | Thermoactinomycetaceae | Thermoactinomyces | Multi-affiliation |
| Cluster_786 | 0.237 | 0.0074 | ** | Firmicutes | Clostridia | Clostridiales | Clostridiaceae 1 | Clostridium sensu stricto 1 | unknown species |
| Cluster_351 | 0.15 | 0.0393 | * | Firmicutes | Clostridia | Clostridiales | Peptostreptococcaceae | Multi-affiliation | Multi-affiliation |
| Cluster_368 | 0.207 | 0.0104 | * | Firmicutes | Erysipelotrichia | Erysipelotrichales | Erysipelotrichaceae | Turicibacter | unknown species |
| Cluster_1782 | 0.217 | 0.0313 | * | Gemmatimonadetes | Gemmatimonadetes | Gemmatimonadales | Gemmatimonadaceae | Gemmatimonas | unknown species |
| Cluster_470 | 0.314 | 0.0015 | ** | Gemmatimonadetes | Gemmatimonadetes | Gemmatimonadales | Gemmatimonadaceae | unknown genus | unknown species |
| Cluster_536 | 0.262 | 0.0132 | * | Gemmatimonadetes | Gemmatimonadetes | Gemmatimonadales | Gemmatimonadaceae | unknown genus | unknown species |
| Cluster_759 | 0.208 | 0.0468 | * | Gemmatimonadetes | Gemmatimonadetes | Gemmatimonadales | Gemmatimonadaceae | unknown genus | unknown species |
| Cluster_4 | 0.205 | 0.0401 | * | Multi-affiliation | Multi-affiliation | Multi-affiliation | Multi-affiliation | Multi-affiliation | Multi-affiliation |
| Cluster_135 | 0.225 | 0.0286 | * | Multi-affiliation | Multi-affiliation | Multi-affiliation | Multi-affiliation | Multi-affiliation | Multi-affiliation |
| Cluster_318 | 0.302 | 0.0021 | ** | Multi-affiliation | Multi-affiliation | Multi-affiliation | Multi-affiliation | Multi-affiliation | Multi-affiliation |
| Cluster_757 | 0.317 | 0.002 | ** | Multi-affiliation | Multi-affiliation | Multi-affiliation | Multi-affiliation | Multi-affiliation | Multi-affiliation |
| Cluster_940 | 0.221 | 0.0322 | * | Multi-affiliation | Multi-affiliation | Multi-affiliation | Multi-affiliation | Multi-affiliation | Multi-affiliation |
| Cluster_213 | 0.284 | 0.005 | ** | Nitrospirae | Nitrospira | Nitrospirales | Nitrospiraceae | Nitrospira | unknown species |
| Cluster_25 | 0.295 | 0.0021 | ** | Proteobacteria | Alphaproteobacteria | Caulobacterales | Caulobacteraceae | Asticcacaulis | Multi-affiliation |
| Cluster_614 | 0.223 | 0.0311 | * | Proteobacteria | Alphaproteobacteria | Caulobacterales | Caulobacteraceae | Caulobacter | Caulobacter sp. |
| Cluster_81 | 0.415 | 0.0001 | *** | Proteobacteria | Alphaproteobacteria | Caulobacterales | Caulobacteraceae | Caulobacter | Multi-affiliation |
| Cluster_720 | 0.21 | 0.0407 | * | Proteobacteria | Alphaproteobacteria | Caulobacterales | Caulobacteraceae | Caulobacter | Multi-affiliation |
| Cluster_156 | 0.39 | 0.0002 | *** | Proteobacteria | Alphaproteobacteria | Caulobacterales | Caulobacteraceae | Phenylobacterium | unknown species |
| Cluster_392 | 0.515 | 0.0001 | *** | Proteobacteria | Alphaproteobacteria | Caulobacterales | Caulobacteraceae | Phenylobacterium | unknown species |
| Cluster_520 | 0.287 | 0.0039 | ** | Proteobacteria | Alphaproteobacteria | Caulobacterales | Caulobacteraceae | unknown genus | Multi-affiliation |
| Cluster_1046 | 0.241 | 0.0045 | ** | Proteobacteria | Alphaproteobacteria | Reyranellales | Reyranellaceae | unknown genus | unknown species |
| Cluster_257 | 0.271 | 0.0023 | ** | Proteobacteria | Alphaproteobacteria | Rhizobiales | Beijerinckiaceae | Methylobacterium | Multi-affiliation |
| Cluster_311 | 0.312 | 0.0017 | ** | Proteobacteria | Alphaproteobacteria | Rhizobiales | Beijerinckiaceae | Methylobacterium | Multi-affiliation |
| Cluster_68 | 0.328 | 0.0012 | ** | Proteobacteria | Alphaproteobacteria | Rhizobiales | Devosiaceae | Devosia | Antarctic bacterium A02 |
| Cluster_364 | 0.261 | 0.0141 | * | Proteobacteria | Alphaproteobacteria | Rhizobiales | Devosiaceae | Devosia | metagenome |
| Cluster_1124 | 0.229 | 0.023 | * | Proteobacteria | Alphaproteobacteria | Rhizobiales | Devosiaceae | Devosia | metagenome |
| Cluster_116 | 0.256 | 0.0157 | * | Proteobacteria | Alphaproteobacteria | Rhizobiales | Devosiaceae | Devosia | Multi-affiliation |
| Cluster_158 | 0.214 | 0.0444 | * | Proteobacteria | Alphaproteobacteria | Rhizobiales | Devosiaceae | Devosia | unknown species |
| Cluster_245 | 0.296 | 0.0046 | ** | Proteobacteria | Alphaproteobacteria | Rhizobiales | Devosiaceae | Devosia | unknown species |
| Cluster_452 | 0.329 | 0.0023 | ** | Proteobacteria | Alphaproteobacteria | Rhizobiales | Devosiaceae | Devosia | unknown species |
| Cluster_508 | 0.301 | 0.0011 | ** | Proteobacteria | Alphaproteobacteria | Rhizobiales | Devosiaceae | Devosia | unknown species |
| Cluster_787 | 0.219 | 0.0376 | * | Proteobacteria | Alphaproteobacteria | Rhizobiales | Hyphomicrobiaceae | Hyphomicrobium | Multi-affiliation |
| Cluster_911 | 0.277 | 0.0048 | ** | Proteobacteria | Alphaproteobacteria | Rhizobiales | Hyphomicrobiaceae | Hyphomicrobium | Multi-affiliation |
| Cluster_810 | 0.212 | 0.0459 | * | Proteobacteria | Alphaproteobacteria | Rhizobiales | Hyphomicrobiaceae | Hyphomicrobium | unknown species |
| Cluster_1132 | 0.237 | 0.026 | * | Proteobacteria | Alphaproteobacteria | Rhizobiales | Hyphomicrobiaceae | Pedomicrobium | metagenome |
| Cluster_650 | 0.309 | 0.0014 | ** | Proteobacteria | Alphaproteobacteria | Rhizobiales | Hyphomicrobiaceae | Pedomicrobium | unknown species |
| Cluster_265 | 0.367 | 0.0005 | *** | Proteobacteria | Alphaproteobacteria | Rhizobiales | Methyloligellaceae | unknown genus | Multi-affiliation |
| Cluster_478 | 0.354 | 0.001 | *** | Proteobacteria | Alphaproteobacteria | Rhizobiales | Methyloligellaceae | unknown genus | Multi-affiliation |
| Cluster_685 | 0.392 | 0.0001 | *** | Proteobacteria | Alphaproteobacteria | Rhizobiales | Methyloligellaceae | unknown genus | unknown species |
| Cluster_52 | 0.295 | 0.005 | ** | Proteobacteria | Alphaproteobacteria | Rhizobiales | Rhizobiaceae | Allorhizobium-Neorhizobium-Pararhizobium-Rhizobium | Multi-affiliation |
| Cluster_86 | 0.388 | 0.0003 | *** | Proteobacteria | Alphaproteobacteria | Rhizobiales | Rhizobiaceae | Allorhizobium-Neorhizobium-Pararhizobium-Rhizobium | Multi-affiliation |
| Cluster_119 | 0.349 | 0.0008 | *** | Proteobacteria | Alphaproteobacteria | Rhizobiales | Rhizobiaceae | Allorhizobium-Neorhizobium-Pararhizobium-Rhizobium | Multi-affiliation |
| Cluster_667 | 0.272 | 0.0055 | ** | Proteobacteria | Alphaproteobacteria | Rhizobiales | Rhizobiaceae | Allorhizobium-Neorhizobium-Pararhizobium-Rhizobium | Multi-affiliation |
| Cluster_142 | 0.202 | 0.0313 | * | Proteobacteria | Alphaproteobacteria | Rhizobiales | Rhizobiaceae | Allorhizobium-Neorhizobium-Pararhizobium-Rhizobium | unknown species |
| Cluster_894 | 0.172 | 0.0109 | * | Proteobacteria | Alphaproteobacteria | Rhizobiales | Rhizobiaceae | Allorhizobium-Neorhizobium-Pararhizobium-Rhizobium | unknown species |
| Cluster_323 | 0.227 | 0.0288 | * | Proteobacteria | Alphaproteobacteria | Rhizobiales | Rhizobiaceae | Aureimonas | Aurantimonas sp. |
| Cluster_133 | 0.474 | 0.0001 | *** | Proteobacteria | Alphaproteobacteria | Rhizobiales | Rhizobiaceae | Mesorhizobium | Multi-affiliation |
| Cluster_310 | 0.4 | 0.0003 | *** | Proteobacteria | Alphaproteobacteria | Rhizobiales | Rhizobiaceae | Mesorhizobium | Multi-affiliation |
| Cluster_241 | 0.348 | 0.0001 | *** | Proteobacteria | Alphaproteobacteria | Rhizobiales | Rhizobiaceae | Neorhizobium | Multi-affiliation |
| Cluster_267 | 0.213 | 0.0388 | * | Proteobacteria | Alphaproteobacteria | Rhizobiales | Rhizobiales Incertae Sedis | unknown genus | unknown species |
| Cluster_925 | 0.257 | 0.0127 | * | Proteobacteria | Alphaproteobacteria | Rhizobiales | Rhizobiales Incertae Sedis | unknown genus | unknown species |
| Cluster_24 | 0.351 | 0.0006 | *** | Proteobacteria | Alphaproteobacteria | Rhizobiales | Xanthobacteraceae | Bradyrhizobium | Multi-affiliation |
| Cluster_198 | 0.22 | 0.0326 | * | Proteobacteria | Alphaproteobacteria | Rhizobiales | Xanthobacteraceae | Bradyrhizobium | unknown species |
| Cluster_107 | 0.495 | 0.0001 | *** | Proteobacteria | Alphaproteobacteria | Rhizobiales | Xanthobacteraceae | Multi-affiliation | Multi-affiliation |
| Cluster_705 | 0.216 | 0.0219 | * | Proteobacteria | Alphaproteobacteria | Rhizobiales | Xanthobacteraceae | Pseudolabrys | Multi-affiliation |
| Cluster_578 | 0.436 | 0.0001 | *** | Proteobacteria | Alphaproteobacteria | Rhizobiales | Xanthobacteraceae | Pseudolabrys | unknown species |
| Cluster_1015 | 0.244 | 0.0188 | * | Proteobacteria | Alphaproteobacteria | Rhizobiales | Xanthobacteraceae | Rhodoplanes | Multi-affiliation |
| Cluster_295 | 0.348 | 0.0005 | *** | Proteobacteria | Alphaproteobacteria | Rhizobiales | Xanthobacteraceae | Rhodoplanes | unknown species |
| Cluster_145 | 0.535 | 0.0001 | *** | Proteobacteria | Alphaproteobacteria | Rhizobiales | Xanthobacteraceae | Rhodopseudomonas | Multi-affiliation |
| Cluster_112 | 0.485 | 0.0001 | *** | Proteobacteria | Alphaproteobacteria | Rhizobiales | Xanthobacteraceae | Tardiphaga | Multi-affiliation |
| Cluster_313 | 0.517 | 0.0001 | *** | Proteobacteria | Alphaproteobacteria | Rhizobiales | Xanthobacteraceae | unknown genus | Multi-affiliation |
| Cluster_404 | 0.353 | 0.0005 | *** | Proteobacteria | Alphaproteobacteria | Rhizobiales | Xanthobacteraceae | unknown genus | Multi-affiliation |
| Cluster_236 | 0.383 | 0.0002 | *** | Proteobacteria | Alphaproteobacteria | Rhizobiales | Xanthobacteraceae | unknown genus | unknown species |
| Cluster_348 | 0.398 | 0.0002 | *** | Proteobacteria | Alphaproteobacteria | Rhizobiales | Xanthobacteraceae | unknown genus | unknown species |
| Cluster_862 | 0.175 | 0.0414 | * | Proteobacteria | Alphaproteobacteria | Rhizobiales | Xanthobacteraceae | unknown genus | unknown species |
| Cluster_875 | 0.3 | 0.0033 | ** | Proteobacteria | Alphaproteobacteria | Rhizobiales | Xanthobacteraceae | unknown genus | unknown species |
| Cluster_1608 | 0.248 | 0.012 | * | Proteobacteria | Alphaproteobacteria | Rhizobiales | Xanthobacteraceae | unknown genus | unknown species |
| Cluster_513 | 0.294 | 0.0025 | ** | Proteobacteria | Alphaproteobacteria | Rhodobacterales | Rhodobacteraceae | Multi-affiliation | Multi-affiliation |
| Cluster_149 | 0.234 | 0.0187 | * | Proteobacteria | Alphaproteobacteria | Rhodobacterales | Rhodobacteraceae | Rhodobacter | Multi-affiliation |
| Cluster_468 | 0.219 | 0.034 | * | Proteobacteria | Alphaproteobacteria | Rhodobacterales | Rhodobacteraceae | Rhodobacter | Multi-affiliation |
| Cluster_131 | 0.215 | 0.0399 | * | Proteobacteria | Alphaproteobacteria | Rhodobacterales | Rhodobacteraceae | Tabrizicola | unknown species |
| Cluster_1004 | 0.208 | 0.0439 | * | Proteobacteria | Alphaproteobacteria | Rhodospirillales | unknown family | unknown genus | metagenome |
| Cluster_1034 | 0.251 | 0.0171 | * | Proteobacteria | Alphaproteobacteria | Rickettsiales | Rickettsiaceae | Candidatus Megaira | unknown species |
| Cluster_449 | 0.269 | 0.0044 | ** | Proteobacteria | Alphaproteobacteria | Rickettsiales | Rickettsiaceae | Rickettsia | Rickettsia endosymbiont of Sitona obsoletus |
| Cluster_490 | 0.273 | 0.008 | ** | Proteobacteria | Alphaproteobacteria | Rickettsiales | SM2D12 | unknown genus | metagenome |
| Cluster_629 | 0.386 | 0.0006 | *** | Proteobacteria | Alphaproteobacteria | Rickettsiales | SM2D12 | unknown genus | metagenome |
| Cluster_944 | 0.236 | 0.018 | * | Proteobacteria | Alphaproteobacteria | Rickettsiales | SM2D12 | unknown genus | unknown species |
| Cluster_945 | 0.194 | 0.0487 | * | Proteobacteria | Alphaproteobacteria | Sphingomonadales | Sphingomonadaceae | Novosphingobium | Multi-affiliation |
| Cluster_155 | 0.36 | 0.0004 | *** | Proteobacteria | Alphaproteobacteria | Sphingomonadales | Sphingomonadaceae | Novosphingobium | unknown species |
| Cluster_1376 | 0.253 | 0.0155 | * | Proteobacteria | Alphaproteobacteria | Sphingomonadales | Sphingomonadaceae | Parablastomonas | unknown species |
| Cluster_1599 | 0.326 | 0.0018 | ** | Proteobacteria | Alphaproteobacteria | Sphingomonadales | Sphingomonadaceae | Sphingomonas | metagenome |
| Cluster_76 | 0.399 | 0.0001 | *** | Proteobacteria | Alphaproteobacteria | Sphingomonadales | Sphingomonadaceae | Sphingomonas | Multi-affiliation |
| Cluster_114 | 0.505 | 0.0001 | *** | Proteobacteria | Alphaproteobacteria | Sphingomonadales | Sphingomonadaceae | Sphingomonas | Multi-affiliation |
| Cluster_191 | 0.285 | 0.0062 | ** | Proteobacteria | Alphaproteobacteria | Sphingomonadales | Sphingomonadaceae | Sphingomonas | Multi-affiliation |
| Cluster_297 | 0.318 | 0.003 | ** | Proteobacteria | Alphaproteobacteria | Sphingomonadales | Sphingomonadaceae | Sphingomonas | Multi-affiliation |
| Cluster_333 | 0.292 | 0.0049 | ** | Proteobacteria | Alphaproteobacteria | Sphingomonadales | Sphingomonadaceae | Sphingomonas | Multi-affiliation |
| Cluster_357 | 0.264 | 0.0043 | ** | Proteobacteria | Alphaproteobacteria | Sphingomonadales | Sphingomonadaceae | Sphingomonas | Multi-affiliation |
| Cluster_480 | 0.327 | 0.001 | *** | Proteobacteria | Alphaproteobacteria | Sphingomonadales | Sphingomonadaceae | Sphingomonas | Multi-affiliation |
| Cluster_523 | 0.208 | 0.0484 | * | Proteobacteria | Alphaproteobacteria | Sphingomonadales | Sphingomonadaceae | Sphingomonas | Multi-affiliation |
| Cluster_884 | 0.291 | 0.0042 | ** | Proteobacteria | Alphaproteobacteria | Sphingomonadales | Sphingomonadaceae | Sphingomonas | Multi-affiliation |
| Cluster_1755 | 0.285 | 0.0039 | ** | Proteobacteria | Alphaproteobacteria | Sphingomonadales | Sphingomonadaceae | Sphingomonas | Multi-affiliation |
| Cluster_602 | 0.209 | 0.0455 | * | Proteobacteria | Alphaproteobacteria | Sphingomonadales | Sphingomonadaceae | Sphingomonas | Sphingomonas sp. |
| Cluster_240 | 0.354 | 0.0005 | *** | Proteobacteria | Alphaproteobacteria | Sphingomonadales | Sphingomonadaceae | Sphingomonas | unknown species |
| Cluster_409 | 0.406 | 0.0001 | *** | Proteobacteria | Alphaproteobacteria | Sphingomonadales | Sphingomonadaceae | Sphingomonas | unknown species |
| Cluster_1150 | 0.193 | 0.05 | * | Proteobacteria | Alphaproteobacteria | Sphingomonadales | Sphingomonadaceae | Sphingomonas | unknown species |
| Cluster_1485 | 0.249 | 0.0119 | * | Proteobacteria | Alphaproteobacteria | Sphingomonadales | Sphingomonadaceae | unknown genus | Multi-affiliation |
| Cluster_400 | 0.294 | 0.0055 | ** | Proteobacteria | Alphaproteobacteria | unknown order | unknown family | unknown genus | unknown species |
| Cluster_718 | 0.308 | 0.0019 | ** | Proteobacteria | Deltaproteobacteria | Bdellovibrionales | Bacteriovoracaceae | Bacteriovorax | unknown species |
| Cluster_1040 | 0.277 | 0.0059 | ** | Proteobacteria | Deltaproteobacteria | Bdellovibrionales | Bdellovibrionaceae | Bdellovibrio | Bdellovibrio sp. |
| Cluster_494 | 0.249 | 0.0126 | * | Proteobacteria | Deltaproteobacteria | Bdellovibrionales | Bdellovibrionaceae | Bdellovibrio | metagenome |
| Cluster_1107 | 0.294 | 0.0021 | ** | Proteobacteria | Deltaproteobacteria | Bdellovibrionales | Bdellovibrionaceae | Bdellovibrio | metagenome |
| Cluster_634 | 0.284 | 0.0062 | ** | Proteobacteria | Deltaproteobacteria | Bdellovibrionales | Bdellovibrionaceae | Bdellovibrio | unknown species |
| Cluster_701 | 0.373 | 0.0001 | *** | Proteobacteria | Deltaproteobacteria | Bdellovibrionales | Bdellovibrionaceae | Bdellovibrio | unknown species |
| Cluster_854 | 0.34 | 0.0005 | *** | Proteobacteria | Deltaproteobacteria | Bdellovibrionales | Bdellovibrionaceae | Bdellovibrio | unknown species |
| Cluster_1507 | 0.248 | 0.0152 | * | Proteobacteria | Deltaproteobacteria | Bdellovibrionales | Bdellovibrionaceae | Bdellovibrio | unknown species |
| Cluster_1588 | 0.256 | 0.0003 | *** | Proteobacteria | Deltaproteobacteria | Bdellovibrionales | Bdellovibrionaceae | Bdellovibrio | unknown species |
| Cluster_553 | 0.234 | 0.023 | * | Proteobacteria | Deltaproteobacteria | Desulfuromonadales | Geobacteraceae | Geobacter | Multi-affiliation |
| Cluster_331 | 0.392 | 0.0001 | *** | Proteobacteria | Deltaproteobacteria | Myxococcales | Haliangiaceae | Haliangium | unknown species |
| Cluster_677 | 0.236 | 0.0243 | * | Proteobacteria | Deltaproteobacteria | Myxococcales | Haliangiaceae | Haliangium | unknown species |
| Cluster_288 | 0.21 | 0.0371 | * | Proteobacteria | Deltaproteobacteria | Myxococcales | Nannocystaceae | Nannocystis | unknown species |
| Cluster_1527 | 0.368 | 0.0003 | *** | Proteobacteria | Deltaproteobacteria | Myxococcales | Polyangiaceae | Pajaroellobacter | unknown species |
| Cluster_659 | 0.387 | 0.0002 | *** | Proteobacteria | Deltaproteobacteria | Oligoflexales | 0319-6G20 | unknown genus | unknown species |
| Cluster_354 | 0.219 | 0.0364 | * | Proteobacteria | Deltaproteobacteria | Oligoflexales | Oligoflexaceae | unknown genus | Multi-affiliation |
| Cluster_9 | 0.208 | 0.045 | * | Proteobacteria | Gammaproteobacteria | Betaproteobacteriales | Burkholderiaceae | Multi-affiliation | Multi-affiliation |
| Cluster_10 | 0.424 | 0.0002 | *** | Proteobacteria | Gammaproteobacteria | Betaproteobacteriales | Burkholderiaceae | Multi-affiliation | Multi-affiliation |
| Cluster_193 | 0.329 | 0.001 | *** | Proteobacteria | Gammaproteobacteria | Betaproteobacteriales | Burkholderiaceae | Multi-affiliation | Multi-affiliation |
| Cluster_779 | 0.227 | 0.0378 | * | Proteobacteria | Gammaproteobacteria | Betaproteobacteriales | Burkholderiaceae | Multi-affiliation | Multi-affiliation |
| Cluster_127 | 0.27 | 0.0077 | ** | Proteobacteria | Gammaproteobacteria | Betaproteobacteriales | Burkholderiaceae | Rhizobacter | Multi-affiliation |
| Cluster_281 | 0.287 | 0.0059 | ** | Proteobacteria | Gammaproteobacteria | Betaproteobacteriales | Burkholderiaceae | Rubrivivax | unknown species |
| Cluster_349 | 0.447 | 0.0001 | *** | Proteobacteria | Gammaproteobacteria | Coxiellales | Coxiellaceae | Coxiella | metagenome |
| Cluster_548 | 0.219 | 0.0364 | * | Proteobacteria | Gammaproteobacteria | Coxiellales | Coxiellaceae | Coxiella | unknown species |
| Cluster_980 | 0.331 | 0.0006 | *** | Proteobacteria | Gammaproteobacteria | Coxiellales | Coxiellaceae | Coxiella | unknown species |
| Cluster_708 | 0.202 | 0.047 | * | Proteobacteria | Gammaproteobacteria | Diplorickettsiales | Diplorickettsiaceae | Aquicella | unknown species |
| Cluster_67 | 0.285 | 0.0046 | ** | Proteobacteria | Gammaproteobacteria | Diplorickettsiales | Diplorickettsiaceae | unknown genus | unknown species |
| Cluster_320 | 0.459 | 0.0001 | *** | Proteobacteria | Gammaproteobacteria | Diplorickettsiales | Diplorickettsiaceae | unknown genus | unknown species |
| Cluster_968 | 0.34 | 0.0005 | *** | Proteobacteria | Gammaproteobacteria | Diplorickettsiales | Diplorickettsiaceae | unknown genus | unknown species |
| Cluster_1041 | 0.232 | 0.0034 | ** | Proteobacteria | Gammaproteobacteria | Diplorickettsiales | Diplorickettsiaceae | unknown genus | unknown species |
| Cluster_124 | 0.243 | 0.0057 | ** | Proteobacteria | Gammaproteobacteria | Enterobacteriales | Enterobacteriaceae | Multi-affiliation | Multi-affiliation |
| Cluster_237 | 0.182 | 0.0005 | *** | Proteobacteria | Gammaproteobacteria | Enterobacteriales | Enterobacteriaceae | Multi-affiliation | Multi-affiliation |
| Cluster_227 | 0.149 | 0.012 | * | Proteobacteria | Gammaproteobacteria | Enterobacteriales | Enterobacteriaceae | Pantoea | Multi-affiliation |
| Cluster_422 | 0.37 | 0.0001 | *** | Proteobacteria | Gammaproteobacteria | Gammaproteobacteria Incertae Sedis | unknown family | Candidatus Berkiella | unknown species |
| Cluster_74 | 0.271 | 0.0081 | ** | Proteobacteria | Gammaproteobacteria | Gammaproteobacteria Incertae Sedis | unknown family | Candidatus Ovatusbacter | unknown species |
| Cluster_251 | 0.214 | 0.0426 | * | Proteobacteria | Gammaproteobacteria | Gammaproteobacteria Incertae Sedis | unknown family | Candidatus Ovatusbacter | unknown species |
| Cluster_167 | 0.534 | 0.0001 | *** | Proteobacteria | Gammaproteobacteria | Legionellales | Legionellaceae | Legionella | Multi-affiliation |
| Cluster_122 | 0.213 | 0.0183 | * | Proteobacteria | Gammaproteobacteria | Pseudomonadales | Moraxellaceae | Acinetobacter | Multi-affiliation |
| Cluster_326 | 0.172 | 0.0395 | * | Proteobacteria | Gammaproteobacteria | Pseudomonadales | Moraxellaceae | Acinetobacter | Multi-affiliation |
| Cluster_501 | 0.183 | 0.0095 | ** | Proteobacteria | Gammaproteobacteria | Pseudomonadales | Moraxellaceae | Acinetobacter | Multi-affiliation |
| Cluster_301 | 0.16 | 0.0497 | * | Proteobacteria | Gammaproteobacteria | Pseudomonadales | Moraxellaceae | Enhydrobacter | Multi-affiliation |
| Cluster_389 | 0.159 | 0.01 | ** | Proteobacteria | Gammaproteobacteria | Pseudomonadales | Moraxellaceae | Enhydrobacter | Multi-affiliation |
| Cluster_205 | 0.336 | 0.0011 | ** | Proteobacteria | Gammaproteobacteria | Pseudomonadales | Moraxellaceae | unknown genus | unknown species |
| Cluster_55 | 0.325 | 0.0017 | ** | Proteobacteria | Gammaproteobacteria | Xanthomonadales | Rhodanobacteraceae | Dokdonella | Multi-affiliation |
| Cluster_336 | 0.238 | 0.0226 | * | Proteobacteria | Gammaproteobacteria | Xanthomonadales | Rhodanobacteraceae | Dokdonella | unknown species |
| Cluster_84 | 0.387 | 0.0001 | *** | Proteobacteria | Gammaproteobacteria | Xanthomonadales | Rhodanobacteraceae | Rhodanobacter | unknown species |
| Cluster_370 | 0.239 | 0.0253 | * | Proteobacteria | Gammaproteobacteria | Xanthomonadales | Rhodanobacteraceae | Rhodanobacter | unknown species |
| Cluster_481 | 0.236 | 0.0224 | * | Proteobacteria | Gammaproteobacteria | Xanthomonadales | Rhodanobacteraceae | Rhodanobacter | unknown species |
| Cluster_952 | 0.244 | 0.0161 | * | Proteobacteria | Gammaproteobacteria | Xanthomonadales | Rhodanobacteraceae | Rhodanobacter | unknown species |
| Cluster_464 | 0.33 | 0.0013 | ** | Proteobacteria | Gammaproteobacteria | Xanthomonadales | Rhodanobacteraceae | Tahibacter | unknown species |
| Cluster_290 | 0.258 | 0.0064 | ** | Proteobacteria | Gammaproteobacteria | Xanthomonadales | Xanthomonadaceae | Arenimonas | unknown species |
| Cluster_294 | 0.222 | 0.0302 | * | Proteobacteria | Gammaproteobacteria | Xanthomonadales | Xanthomonadaceae | Arenimonas | unknown species |
| Cluster_540 | 0.41 | 0.0001 | *** | Proteobacteria | Gammaproteobacteria | Xanthomonadales | Xanthomonadaceae | Arenimonas | unknown species |
| Cluster_1240 | 0.255 | 0.0095 | ** | Proteobacteria | Gammaproteobacteria | Xanthomonadales | Xanthomonadaceae | Arenimonas | unknown species |
|  |  |  |  |  |  |  |  |  |  |
| **Bacteria - Modern Cultivars** | | |  |  |  |  |  |  |  |
| Cluster_997 | 0.168 | 0.0359 | * | Actinobacteria | Actinobacteria | Streptomycetales | Streptomycetaceae | Kitasatospora | unknown species |
| Cluster_20 | 0.317 | 0.0008 | *** | Actinobacteria | Actinobacteria | Streptomycetales | Streptomycetaceae | Streptomyces | Multi-affiliation |
| Cluster_90 | 0.378 | 0.0001 | *** | Actinobacteria | Actinobacteria | Streptomycetales | Streptomycetaceae | Streptomyces | Multi-affiliation |
| Cluster_632 | 0.214 | 0.0001 | *** | Actinobacteria | Actinobacteria | Streptomycetales | Streptomycetaceae | Streptomyces | Multi-affiliation |
| Cluster_504 | 0.215 | 0.0364 | * | Bacteroidetes | Bacteroidia | Chitinophagales | Chitinophagaceae | unknown genus | unknown species |
| Cluster_761 | 0.247 | 0.014 | * | Bacteroidetes | Bacteroidia | Chitinophagales | Chitinophagaceae | unknown genus | unknown species |
| Cluster_1639 | 0.231 | 0.0278 | * | Bacteroidetes | Bacteroidia | Chitinophagales | Chitinophagaceae | unknown genus | unknown species |
| Cluster_477 | 0.217 | 0.0362 | * | Bacteroidetes | Bacteroidia | Cytophagales | Hymenobacteraceae | Adhaeribacter | Adhaeribacter sp. |
| Cluster_483 | 0.213 | 0.0445 | * | Bacteroidetes | Bacteroidia | Cytophagales | Microscillaceae | Ohtaekwangia | metagenome |
| Cluster_455 | 0.203 | 0.005 | ** | Bacteroidetes | Bacteroidia | Cytophagales | Microscillaceae | Ohtaekwangia | unknown species |
| Cluster_524 | 0.263 | 0.0091 | ** | Bacteroidetes | Bacteroidia | Cytophagales | Microscillaceae | unknown genus | metagenome |
| Cluster_342 | 0.415 | 0.0001 | *** | Bacteroidetes | Bacteroidia | Cytophagales | Microscillaceae | unknown genus | unknown species |
| Cluster_495 | 0.346 | 0.0004 | *** | Bacteroidetes | Bacteroidia | Cytophagales | Microscillaceae | unknown genus | unknown species |
| Cluster_567 | 0.232 | 0.0186 | * | Bacteroidetes | Bacteroidia | Cytophagales | Microscillaceae | unknown genus | unknown species |
| Cluster_623 | 0.345 | 0.0003 | *** | Bacteroidetes | Bacteroidia | Cytophagales | Microscillaceae | unknown genus | unknown species |
| Cluster_1085 | 0.32 | 0.0008 | *** | Bacteroidetes | Bacteroidia | Cytophagales | Microscillaceae | unknown genus | unknown species |
| Cluster_1333 | 0.206 | 0.0335 | * | Bacteroidetes | Bacteroidia | Cytophagales | Microscillaceae | unknown genus | unknown species |
| Cluster_51 | 0.359 | 0.0001 | *** | Bacteroidetes | Bacteroidia | Flavobacteriales | Flavobacteriaceae | Flavobacterium | cf. Chryseobacterium sp. UOF CM895 |
| Cluster_232 | 0.206 | 0.0493 | * | Bacteroidetes | Bacteroidia | Flavobacteriales | Flavobacteriaceae | Flavobacterium | Flavobacterium hauense |
| Cluster_15 | 0.378 | 0.0001 | *** | Bacteroidetes | Bacteroidia | Flavobacteriales | Flavobacteriaceae | Flavobacterium | Flavobacterium sp. |
| Cluster_196 | 0.312 | 0.0001 | *** | Bacteroidetes | Bacteroidia | Flavobacteriales | Flavobacteriaceae | Flavobacterium | Flavobacterium sp. |
| Cluster_441 | 0.273 | 0.003 | ** | Bacteroidetes | Bacteroidia | Flavobacteriales | Flavobacteriaceae | Flavobacterium | Flavobacterium sp. |
| Cluster_454 | 0.278 | 0.0004 | *** | Bacteroidetes | Bacteroidia | Flavobacteriales | Flavobacteriaceae | Flavobacterium | Flavobacterium sp. |
| Cluster_770 | 0.429 | 0.0001 | *** | Bacteroidetes | Bacteroidia | Flavobacteriales | Flavobacteriaceae | Flavobacterium | Flavobacterium sp. |
| Cluster_813 | 0.168 | 0.0218 | * | Bacteroidetes | Bacteroidia | Flavobacteriales | Flavobacteriaceae | Flavobacterium | Flavobacterium sp. |
| Cluster_509 | 0.363 | 0.0001 | *** | Bacteroidetes | Bacteroidia | Flavobacteriales | Flavobacteriaceae | Flavobacterium | metagenome |
| Cluster_5 | 0.284 | 0.0052 | ** | Bacteroidetes | Bacteroidia | Flavobacteriales | Flavobacteriaceae | Flavobacterium | Multi-affiliation |
| Cluster_7 | 0.455 | 0.0001 | *** | Bacteroidetes | Bacteroidia | Flavobacteriales | Flavobacteriaceae | Flavobacterium | Multi-affiliation |
| Cluster_8 | 0.465 | 0.0001 | *** | Bacteroidetes | Bacteroidia | Flavobacteriales | Flavobacteriaceae | Flavobacterium | Multi-affiliation |
| Cluster_11 | 0.458 | 0.0001 | *** | Bacteroidetes | Bacteroidia | Flavobacteriales | Flavobacteriaceae | Flavobacterium | Multi-affiliation |
| Cluster_78 | 0.304 | 0.0001 | *** | Bacteroidetes | Bacteroidia | Flavobacteriales | Flavobacteriaceae | Flavobacterium | Multi-affiliation |
| Cluster_92 | 0.223 | 0.0281 | * | Bacteroidetes | Bacteroidia | Flavobacteriales | Flavobacteriaceae | Flavobacterium | Multi-affiliation |
| Cluster_110 | 0.395 | 0.0001 | *** | Bacteroidetes | Bacteroidia | Flavobacteriales | Flavobacteriaceae | Flavobacterium | Multi-affiliation |
| Cluster_195 | 0.191 | 0.0006 | *** | Bacteroidetes | Bacteroidia | Flavobacteriales | Flavobacteriaceae | Flavobacterium | Multi-affiliation |
| Cluster_217 | 0.26 | 0.0009 | *** | Bacteroidetes | Bacteroidia | Flavobacteriales | Flavobacteriaceae | Flavobacterium | Multi-affiliation |
| Cluster_234 | 0.369 | 0.0001 | *** | Bacteroidetes | Bacteroidia | Flavobacteriales | Flavobacteriaceae | Flavobacterium | Multi-affiliation |
| Cluster_248 | 0.364 | 0.0004 | *** | Bacteroidetes | Bacteroidia | Flavobacteriales | Flavobacteriaceae | Flavobacterium | Multi-affiliation |
| Cluster_292 | 0.297 | 0.0032 | ** | Bacteroidetes | Bacteroidia | Flavobacteriales | Flavobacteriaceae | Flavobacterium | Multi-affiliation |
| Cluster_385 | 0.304 | 0.0033 | ** | Bacteroidetes | Bacteroidia | Flavobacteriales | Flavobacteriaceae | Flavobacterium | Multi-affiliation |
| Cluster_388 | 0.244 | 0.0155 | * | Bacteroidetes | Bacteroidia | Flavobacteriales | Flavobacteriaceae | Flavobacterium | Multi-affiliation |
| Cluster_437 | 0.254 | 0.0013 | ** | Bacteroidetes | Bacteroidia | Flavobacteriales | Flavobacteriaceae | Flavobacterium | Multi-affiliation |
| Cluster_638 | 0.317 | 0.0003 | *** | Bacteroidetes | Bacteroidia | Flavobacteriales | Flavobacteriaceae | Flavobacterium | Multi-affiliation |
| Cluster_751 | 0.232 | 0.0251 | * | Bacteroidetes | Bacteroidia | Flavobacteriales | Flavobacteriaceae | Flavobacterium | Multi-affiliation |
| Cluster_792 | 0.288 | 0.0047 | ** | Bacteroidetes | Bacteroidia | Flavobacteriales | Flavobacteriaceae | Flavobacterium | Multi-affiliation |
| Cluster_849 | 0.271 | 0.0055 | ** | Bacteroidetes | Bacteroidia | Flavobacteriales | Flavobacteriaceae | Flavobacterium | Multi-affiliation |
| Cluster_1043 | 0.3 | 0.0015 | ** | Bacteroidetes | Bacteroidia | Flavobacteriales | Flavobacteriaceae | Flavobacterium | Multi-affiliation |
| Cluster_1157 | 0.325 | 0.001 | *** | Bacteroidetes | Bacteroidia | Flavobacteriales | Flavobacteriaceae | Flavobacterium | Multi-affiliation |
| Cluster_1522 | 0.194 | 0.0273 | * | Bacteroidetes | Bacteroidia | Flavobacteriales | Flavobacteriaceae | Flavobacterium | Multi-affiliation |
| Cluster_1753 | 0.272 | 0.0035 | ** | Bacteroidetes | Bacteroidia | Flavobacteriales | Flavobacteriaceae | Flavobacterium | Multi-affiliation |
| Cluster_41 | 0.214 | 0.0416 | * | Bacteroidetes | Bacteroidia | Flavobacteriales | Flavobacteriaceae | Flavobacterium | unknown species |
| Cluster_130 | 0.42 | 0.0001 | *** | Bacteroidetes | Bacteroidia | Flavobacteriales | Flavobacteriaceae | Flavobacterium | unknown species |
| Cluster_242 | 0.197 | 0.0186 | * | Bacteroidetes | Bacteroidia | Flavobacteriales | Flavobacteriaceae | Flavobacterium | unknown species |
| Cluster_387 | 0.341 | 0.0006 | *** | Bacteroidetes | Bacteroidia | Flavobacteriales | Flavobacteriaceae | Flavobacterium | unknown species |
| Cluster_654 | 0.344 | 0.0003 | *** | Bacteroidetes | Bacteroidia | Flavobacteriales | Flavobacteriaceae | Flavobacterium | unknown species |
| Cluster_767 | 0.229 | 0.0168 | * | Bacteroidetes | Bacteroidia | Flavobacteriales | Flavobacteriaceae | Flavobacterium | unknown species |
| Cluster_800 | 0.237 | 0.0146 | * | Bacteroidetes | Bacteroidia | Flavobacteriales | Flavobacteriaceae | Flavobacterium | unknown species |
| Cluster_1016 | 0.153 | 0.0051 | ** | Bacteroidetes | Bacteroidia | Flavobacteriales | Flavobacteriaceae | Flavobacterium | unknown species |
| Cluster_1412 | 0.255 | 0.0062 | ** | Bacteroidetes | Bacteroidia | Flavobacteriales | Flavobacteriaceae | Flavobacterium | unknown species |
| Cluster_1268 | 0.233 | 0.0227 | * | Bacteroidetes | Bacteroidia | Flavobacteriales | NS9 marine group | unknown genus | metagenome |
| Cluster_1018 | 0.234 | 0.0113 | * | Bacteroidetes | Bacteroidia | Sphingobacteriales | Sphingobacteriaceae | Mucilaginibacter | Multi-affiliation |
| Cluster_725 | 0.239 | 0.0085 | ** | Bacteroidetes | Bacteroidia | Sphingobacteriales | Sphingobacteriaceae | Pedobacter | unknown species |
| Cluster_1427 | 0.223 | 0.0215 | * | Bacteroidetes | Bacteroidia | Sphingobacteriales | Sphingobacteriaceae | Solitalea | unknown species |
| Cluster_262 | 0.441 | 0.0001 | *** | Bacteroidetes | Bacteroidia+EE33:E43 | Cytophagales | Microscillaceae | unknown genus | unknown species |
| Cluster_1264 | 0.432 | 0.0001 | *** | Fibrobacteres | Fibrobacteria | Fibrobacterales | Fibrobacteraceae | possible genus 04 | Multi-affiliation |
| Cluster_293 | 0.274 | 0.0007 | *** | Fibrobacteres | Fibrobacteria | Fibrobacterales | Fibrobacteraceae | possible genus 04 | unknown species |
| Cluster_244 | 0.225 | 0.0319 | * | Firmicutes | Bacilli | Bacillales | Bacillaceae | Bacillus | Multi-affiliation |
| Cluster_752 | 0.417 | 0.0001 | *** | Firmicutes | Bacilli | Bacillales | Paenibacillaceae | Paenibacillus | bacterium B1031 |
| Cluster_99 | 0.301 | 0.0028 | ** | Firmicutes | Bacilli | Bacillales | Paenibacillaceae | Paenibacillus | Multi-affiliation |
| Cluster_372 | 0.28 | 0.008 | ** | Firmicutes | Bacilli | Bacillales | Paenibacillaceae | Paenibacillus | Multi-affiliation |
| Cluster_1302 | 0.248 | 0.0174 | * | Firmicutes | Bacilli | Bacillales | Paenibacillaceae | Paenibacillus | Multi-affiliation |
| Cluster_1304 | 0.219 | 0.0345 | * | Firmicutes | Bacilli | Bacillales | Paenibacillaceae | Paenibacillus | Multi-affiliation |
| Cluster_563 | 0.387 | 0.0001 | *** | Firmicutes | Bacilli | Bacillales | Paenibacillaceae | Paenibacillus | Paenibacillus aceris |
| Cluster_412 | 0.468 | 0.0001 | *** | Firmicutes | Bacilli | Bacillales | Paenibacillaceae | Paenibacillus | Paenibacillus alginolyticus |
| Cluster_874 | 0.426 | 0.0001 | *** | Firmicutes | Bacilli | Bacillales | Paenibacillaceae | Paenibacillus | Paenibacillus chondroitinus |
| Cluster_391 | 0.295 | 0.0037 | ** | Firmicutes | Bacilli | Bacillales | Paenibacillaceae | Paenibacillus | Paenibacillus sp. |
| Cluster_681 | 0.447 | 0.0001 | *** | Firmicutes | Bacilli | Bacillales | Paenibacillaceae | Paenibacillus | Paenibacillus sp. |
| Cluster_785 | 0.485 | 0.0001 | *** | Multi-affiliation | Multi-affiliation | Multi-affiliation | Multi-affiliation | Multi-affiliation | Multi-affiliation |
| Cluster_791 | 0.237 | 0.0265 | * | Proteobacteria | Alphaproteobacteria | Caulobacterales | Caulobacteraceae | Brevundimonas | Multi-affiliation |
| Cluster_382 | 0.244 | 0.0206 | * | Proteobacteria | Alphaproteobacteria | Dongiales | Dongiaceae | Dongia | Multi-affiliation |
| Cluster_337 | 0.249 | 0.0186 | * | Proteobacteria | Alphaproteobacteria | Dongiales | Dongiaceae | Dongia | unknown species |
| Cluster_403 | 0.27 | 0.0092 | ** | Proteobacteria | Alphaproteobacteria | Micropepsales | Micropepsaceae | unknown genus | Multi-affiliation |
| Cluster_1250 | 0.228 | 0.016 | * | Proteobacteria | Alphaproteobacteria | Rhizobiales | A0839 | unknown genus | unknown species |
| Cluster_870 | 0.249 | 0.0142 | * | Proteobacteria | Alphaproteobacteria | Rhizobiales | Beijerinckiaceae | Methylobacterium | Methylobacterium sp. |
| Cluster_726 | 0.207 | 0.0332 | * | Proteobacteria | Alphaproteobacteria | Rhizobiales | Devosiaceae | Devosia | Multi-affiliation |
| Cluster_147 | 0.206 | 0.0498 | * | Proteobacteria | Alphaproteobacteria | Rhizobiales | Devosiaceae | Devosia | unknown species |
| Cluster_544 | 0.279 | 0.0048 | ** | Proteobacteria | Alphaproteobacteria | Rhizobiales | Rhizobiaceae | Allorhizobium-Neorhizobium-Pararhizobium-Rhizobium | Multi-affiliation |
| Cluster_749 | 0.192 | 0.0262 | * | Proteobacteria | Alphaproteobacteria | Rhizobiales | Rhizobiaceae | Ochrobactrum | Multi-affiliation |
| Cluster_956 | 0.322 | 0.0021 | ** | Proteobacteria | Alphaproteobacteria | Rickettsiales | Mitochondria | unknown genus | Gossypium hirsutum (cotton) |
| Cluster_1082 | 0.304 | 0.0051 | ** | Proteobacteria | Alphaproteobacteria | Rickettsiales | Mitochondria | unknown genus | Prunus armeniaca (apricot) |
| Cluster_868 | 0.259 | 0.0146 | * | Proteobacteria | Alphaproteobacteria | Sphingomonadales | Sphingomonadaceae | Sphingomonas | unknown species |
| Cluster_605 | 0.395 | 0.0002 | *** | Proteobacteria | Deltaproteobacteria | Bdellovibrionales | Bacteriovoracaceae | Bacteriovorax | Bacteriovorax sp. |
| Cluster_1645 | 0.24 | 0.0187 | * | Proteobacteria | Deltaproteobacteria | Bdellovibrionales | Bacteriovoracaceae | Peredibacter | Bacteriovorax sp. |
| Cluster_570 | 0.25 | 0.001 | *** | Proteobacteria | Deltaproteobacteria | Myxococcales | Haliangiaceae | Haliangium | metagenome |
| Cluster_502 | 0.261 | 0.0101 | * | Proteobacteria | Deltaproteobacteria | Myxococcales | Haliangiaceae | Haliangium | unknown species |
| Cluster_972 | 0.223 | 0.0201 | * | Proteobacteria | Deltaproteobacteria | Myxococcales | Haliangiaceae | Haliangium | unknown species |
| Cluster_1013 | 0.307 | 0.0019 | ** | Proteobacteria | Deltaproteobacteria | Myxococcales | Myxococcaceae | Corallococcus | Multi-affiliation |
| Cluster_419 | 0.399 | 0.0001 | *** | Proteobacteria | Deltaproteobacteria | Myxococcales | Phaselicystidaceae | Phaselicystis | metagenome |
| Cluster_2189 | 0.365 | 0.0002 | *** | Proteobacteria | Deltaproteobacteria | Myxococcales | Polyangiaceae | Polyangium | metagenome |
| Cluster_229 | 0.332 | 0.0012 | ** | Proteobacteria | Deltaproteobacteria | Myxococcales | Sandaracinaceae | unknown genus | unknown species |
| Cluster_312 | 0.226 | 0.0342 | * | Proteobacteria | Deltaproteobacteria | Myxococcales | Sandaracinaceae | unknown genus | unknown species |
| Cluster_209 | 0.369 | 0.0001 | *** | Proteobacteria | Gammaproteobacteria | Aeromonadales | Aeromonadaceae | Aeromonas | Multi-affiliation |
| Cluster_604 | 0.358 | 0.0002 | *** | Proteobacteria | Gammaproteobacteria | Betaproteobacteriales | Burkholderiaceae | Acidovorax | Multi-affiliation |
| Cluster_1779 | 0.363 | 0.0005 | *** | Proteobacteria | Gammaproteobacteria | Betaproteobacteriales | Burkholderiaceae | Acidovorax | unknown species |
| Cluster_1292 | 0.219 | 0.0134 | * | Proteobacteria | Gammaproteobacteria | Betaproteobacteriales | Burkholderiaceae | Aquabacterium | metagenome |
| Cluster_1125 | 0.232 | 0.0291 | * | Proteobacteria | Gammaproteobacteria | Betaproteobacteriales | Burkholderiaceae | Burkholderia-Caballeronia-Paraburkholderia | unknown species |
| Cluster_69 | 0.428 | 0.0001 | *** | Proteobacteria | Gammaproteobacteria | Betaproteobacteriales | Burkholderiaceae | Comamonas | Multi-affiliation |
| Cluster_381 | 0.306 | 0.0012 | ** | Proteobacteria | Gammaproteobacteria | Betaproteobacteriales | Burkholderiaceae | Ideonella | Ideonella sp. |
| Cluster_200 | 0.26 | 0.0097 | ** | Proteobacteria | Gammaproteobacteria | Betaproteobacteriales | Burkholderiaceae | Ideonella | Multi-affiliation |
| Cluster_809 | 0.296 | 0.0045 | ** | Proteobacteria | Gammaproteobacteria | Betaproteobacteriales | Burkholderiaceae | Massilia | unknown species |
| Cluster_994 | 0.255 | 0.0172 | * | Proteobacteria | Gammaproteobacteria | Betaproteobacteriales | Burkholderiaceae | Massilia | unknown species |
| Cluster_32 | 0.336 | 0.0009 | *** | Proteobacteria | Gammaproteobacteria | Betaproteobacteriales | Burkholderiaceae | Multi-affiliation | Multi-affiliation |
| Cluster_57 | 0.426 | 0.0001 | *** | Proteobacteria | Gammaproteobacteria | Betaproteobacteriales | Burkholderiaceae | Multi-affiliation | Multi-affiliation |
| Cluster_61 | 0.238 | 0.013 | * | Proteobacteria | Gammaproteobacteria | Betaproteobacteriales | Burkholderiaceae | Multi-affiliation | Multi-affiliation |
| Cluster_77 | 0.396 | 0.0001 | *** | Proteobacteria | Gammaproteobacteria | Betaproteobacteriales | Burkholderiaceae | Multi-affiliation | Multi-affiliation |
| Cluster_826 | 0.266 | 0.0066 | ** | Proteobacteria | Gammaproteobacteria | Betaproteobacteriales | Burkholderiaceae | Multi-affiliation | Multi-affiliation |
| Cluster_969 | 0.316 | 0.0005 | *** | Proteobacteria | Gammaproteobacteria | Betaproteobacteriales | Burkholderiaceae | Multi-affiliation | Multi-affiliation |
| Cluster_1803 | 0.314 | 0.0027 | ** | Proteobacteria | Gammaproteobacteria | Betaproteobacteriales | Burkholderiaceae | Multi-affiliation | Multi-affiliation |
| Cluster_624 | 0.354 | 0.0005 | *** | Proteobacteria | Gammaproteobacteria | Betaproteobacteriales | Burkholderiaceae | Paucibacter | Multi-affiliation |
| Cluster_334 | 0.212 | 0.0264 | * | Proteobacteria | Gammaproteobacteria | Betaproteobacteriales | Burkholderiaceae | Pseudorhodoferax | Multi-affiliation |
| Cluster_448 | 0.217 | 0.0374 | * | Proteobacteria | Gammaproteobacteria | Betaproteobacteriales | Burkholderiaceae | Rhizobacter | Multi-affiliation |
| Cluster_146 | 0.222 | 0.0316 | * | Proteobacteria | Gammaproteobacteria | Betaproteobacteriales | Burkholderiaceae | unknown genus | Multi-affiliation |
| Cluster_866 | 0.278 | 0.0052 | ** | Proteobacteria | Gammaproteobacteria | Betaproteobacteriales | Burkholderiaceae | unknown genus | unknown species |
| Cluster_153 | 0.406 | 0.0001 | *** | Proteobacteria | Gammaproteobacteria | Betaproteobacteriales | Burkholderiaceae | Variovorax | Multi-affiliation |
| Cluster_1061 | 0.259 | 0.0121 | * | Proteobacteria | Gammaproteobacteria | CCD24 | unknown family | unknown genus | Multi-affiliation |
| Cluster_95 | 0.613 | 0.0001 | *** | Proteobacteria | Gammaproteobacteria | Cellvibrionales | Cellvibrionaceae | Cellvibrio | Multi-affiliation |
| Cluster_141 | 0.285 | 0.0001 | *** | Proteobacteria | Gammaproteobacteria | Cellvibrionales | Cellvibrionaceae | Cellvibrio | Multi-affiliation |
| Cluster_867 | 0.341 | 0.0004 | *** | Proteobacteria | Gammaproteobacteria | Cellvibrionales | Cellvibrionaceae | Cellvibrio | Multi-affiliation |
| Cluster_374 | 0.258 | 0.0144 | * | Proteobacteria | Gammaproteobacteria | Cellvibrionales | Cellvibrionaceae | Cellvibrio | unknown species |
| Cluster_2084 | 0.44 | 0.0001 | *** | Proteobacteria | Gammaproteobacteria | Cellvibrionales | Cellvibrionaceae | Cellvibrio | unknown species |
| Cluster_379 | 0.256 | 0.0109 | * | Proteobacteria | Gammaproteobacteria | Enterobacteriales | Enterobacteriaceae | Enterobacter | unknown species |
| Cluster_22 | 0.219 | 0.0096 | ** | Proteobacteria | Gammaproteobacteria | Enterobacteriales | Enterobacteriaceae | Erwinia | Multi-affiliation |
| Cluster_28 | 0.335 | 0.0001 | *** | Proteobacteria | Gammaproteobacteria | Enterobacteriales | Enterobacteriaceae | Erwinia | Multi-affiliation |
| Cluster_14 | 0.252 | 0.0096 | ** | Proteobacteria | Gammaproteobacteria | Enterobacteriales | Enterobacteriaceae | Multi-affiliation | Multi-affiliation |
| Cluster_18 | 0.231 | 0.0156 | * | Proteobacteria | Gammaproteobacteria | Enterobacteriales | Enterobacteriaceae | Multi-affiliation | Multi-affiliation |
| Cluster_70 | 0.293 | 0.002 | ** | Proteobacteria | Gammaproteobacteria | Enterobacteriales | Enterobacteriaceae | Multi-affiliation | Multi-affiliation |
| Cluster_585 | 0.233 | 0.0074 | ** | Proteobacteria | Gammaproteobacteria | Enterobacteriales | Enterobacteriaceae | Multi-affiliation | Multi-affiliation |
| Cluster_1051 | 0.278 | 0.008 | ** | Proteobacteria | Gammaproteobacteria | Enterobacteriales | Enterobacteriaceae | Multi-affiliation | Multi-affiliation |
| Cluster_499 | 0.297 | 0.0013 | ** | Proteobacteria | Gammaproteobacteria | Enterobacteriales | Enterobacteriaceae | Rahnella | Multi-affiliation |
| Cluster_46 | 0.231 | 0.0278 | * | Proteobacteria | Gammaproteobacteria | Gammaproteobacteria Incertae Sedis | unknown family | Acidibacter | unknown species |
| Cluster_152 | 0.236 | 0.0231 | * | Proteobacteria | Gammaproteobacteria | Gammaproteobacteria Incertae Sedis | unknown family | Acidibacter | unknown species |
| Cluster_511 | 0.222 | 0.0336 | * | Proteobacteria | Gammaproteobacteria | Gammaproteobacteria Incertae Sedis | unknown family | Candidatus Berkiella | unknown species |
| Cluster_620 | 0.234 | 0.0268 | * | Proteobacteria | Gammaproteobacteria | Multi-affiliation | Multi-affiliation | Multi-affiliation | Multi-affiliation |
| Cluster_463 | 0.328 | 0.002 | ** | Proteobacteria | Gammaproteobacteria | Oceanospirillales | Halomonadaceae | Carnimonas | unknown species |
| Cluster_479 | 0.215 | 0.0401 | * | Proteobacteria | Gammaproteobacteria | Pseudomonadales | Moraxellaceae | Alkanindiges | unknown species |
| Cluster_3 | 0.561 | 0.0001 | *** | Proteobacteria | Gammaproteobacteria | Pseudomonadales | Pseudomonadaceae | Pseudomonas | Multi-affiliation |
| Cluster_34 | 0.305 | 0.0023 | ** | Proteobacteria | Gammaproteobacteria | Pseudomonadales | Pseudomonadaceae | Pseudomonas | Multi-affiliation |
| Cluster_72 | 0.492 | 0.0001 | *** | Proteobacteria | Gammaproteobacteria | Pseudomonadales | Pseudomonadaceae | Pseudomonas | Multi-affiliation |
| Cluster_327 | 0.364 | 0.0001 | *** | Proteobacteria | Gammaproteobacteria | Pseudomonadales | Pseudomonadaceae | Pseudomonas | Multi-affiliation |
| Cluster_569 | 0.126 | 0.0314 | * | Proteobacteria | Gammaproteobacteria | Pseudomonadales | Pseudomonadaceae | Pseudomonas | Multi-affiliation |
| Cluster_671 | 0.298 | 0.0011 | ** | Proteobacteria | Gammaproteobacteria | Pseudomonadales | Pseudomonadaceae | Pseudomonas | Multi-affiliation |
| Cluster_676 | 0.404 | 0.0001 | *** | Proteobacteria | Gammaproteobacteria | Pseudomonadales | Pseudomonadaceae | Pseudomonas | Multi-affiliation |
| Cluster_820 | 0.336 | 0.0001 | *** | Proteobacteria | Gammaproteobacteria | Pseudomonadales | Pseudomonadaceae | Pseudomonas | Multi-affiliation |
| Cluster_1243 | 0.232 | 0.0276 | * | Proteobacteria | Gammaproteobacteria | Pseudomonadales | Pseudomonadaceae | Pseudomonas | Multi-affiliation |
| Cluster_386 | 0.441 | 0.0001 | *** | Proteobacteria | Gammaproteobacteria | Pseudomonadales | Pseudomonadaceae | Pseudomonas | Pseudomonas sp. |
| Cluster_574 | 0.284 | 0.0037 | ** | Proteobacteria | Gammaproteobacteria | Pseudomonadales | Pseudomonadaceae | Pseudomonas | Pseudomonas sp. |
| Cluster_1138 | 0.318 | 0.0017 | ** | Proteobacteria | Gammaproteobacteria | Pseudomonadales | Pseudomonadaceae | Pseudomonas | Pseudomonas sp. |
| Cluster_1641 | 0.424 | 0.0001 | *** | Proteobacteria | Gammaproteobacteria | Pseudomonadales | Pseudomonadaceae | Pseudomonas | Spirillum pleomorphum |
| Cluster_143 | 0.465 | 0.0001 | *** | Proteobacteria | Gammaproteobacteria | Pseudomonadales | Pseudomonadaceae | Pseudomonas | unknown species |
| Cluster_291 | 0.163 | 0.0051 | ** | Proteobacteria | Gammaproteobacteria | Pseudomonadales | Pseudomonadaceae | Pseudomonas | unknown species |
| Cluster_423 | 0.262 | 0.0108 | * | Proteobacteria | Gammaproteobacteria | Pseudomonadales | Pseudomonadaceae | Pseudomonas | unknown species |
| Cluster_466 | 0.26 | 0.0135 | * | Proteobacteria | Gammaproteobacteria | Pseudomonadales | Pseudomonadaceae | Pseudomonas | unknown species |
| Cluster_1174 | 0.242 | 0.0243 | * | Proteobacteria | Gammaproteobacteria | R7C24 | unknown family | unknown genus | Multi-affiliation |
| Cluster_453 | 0.255 | 0.0122 | * | Proteobacteria | Gammaproteobacteria | Xanthomonadales | Rhodanobacteraceae | Tahibacter | unknown species |
| Cluster_39 | 0.301 | 0.0022 | ** | Proteobacteria | Gammaproteobacteria | Xanthomonadales | Xanthomonadaceae | Multi-affiliation | Multi-affiliation |
| Cluster_484 | 0.231 | 0.0292 | * | Proteobacteria | Gammaproteobacteria | Xanthomonadales | Xanthomonadaceae | Multi-affiliation | Multi-affiliation |
| Cluster_1338 | 0.225 | 0.03 | * | Spirochaetes | Spirochaetia | Spirochaetales | Spirochaetaceae | Salinispira | metagenome |
| Cluster_399 | 0.432 | 0.0001 | *** | Spirochaetes | Spirochaetia | Spirochaetales | Spirochaetaceae | Spirochaeta 2 | metagenome |
| Cluster_393 | 0.251 | 0.0071 | ** | Spirochaetes | Spirochaetia | Spirochaetales | Spirochaetaceae | Spirochaeta 2 | unknown species |
